# Modeling the Tradeoff Between Water Loss, Chlorine Residuals, and Trihalomethanes in Rural Appalachia, USA

**DOI:** 10.3390/w17213138

**Published:** 2025-10-31

**Authors:** George Fordjour, Yogesh Gautam, Lindell Ormsbee, Scott Yost, Jason Unrine

**Affiliations:** 1 Department of Civil Engineering, University of Kentucky, Lexington, KY 40506, USA;; 2 Public Works Division, Saint Paul, MN 55102, USA;; 3 Department of Plant and Soil Sciences, University of Kentucky, Lexington, KY 40506, USA;; 4 Kentucky Water Research Institute, University of Kentucky, Lexington, KY 40506, USA

**Keywords:** water quality modeling, hydraulic modeling, water loss optimization, chlorine residual modeling, TTHM modeling, Appalachian water utilities, KYPIPE, EPANET, water distribution systems

## Abstract

Small rural water utilities in the Appalachia region of the US often experience extreme water loss while struggling to maintain water quality compliance. This study quantifies the impact of reducing water loss on distribution system water quality in Martin County, Kentucky. Hydraulic and water quality models were developed, calibrated, and validated using EPANET for chlorine residuals and KYPIPE for trihalomethane (TTHM) formation. The models evaluated water loss reduction scenarios ranging from the current 70% to the industry target of 15%. Results showed that lowering water loss increased residence times, causing chlorine residual declines of 22–68%, with one site falling to the 0.2 mg/L threshold. TTHM concentrations increased by 12–18% in winter–spring and 26–44% in summer–fall, with two sites exceeding the individual 0.080 mg/L maximum contaminant level. These novel findings indicate that reducing water loss can unintentionally degrade water quality, underscoring the need for integrated planning. Recommended mitigation strategies include seasonal operational adjustments, water source and TTHM precursor management, optimized tank management, targeted flushing, and phased infrastructure upgrades. The modeling framework developed offers potential for broader application in other rural systems facing similar challenges.

## 1. Introduction

Water utilities encompass vital community infrastructure that facilitates the reliable delivery of safe, clean water to many homes, businesses, and essential services. According to the World Health Organization (WHO), access to safe drinking water is not merely a convenience but a cornerstone of public health, playing a crucial role in disease prevention and overall well-being [1]. This highlights the crucial importance of maintaining well-functioning water utilities at all levels. In the United States, approximately 90% of the population relies on public water utilities for their drinking water, with over 154,000 such systems in operation [2,3]. In Kentucky, this figure is even higher, with about 95% of residents receiving their drinking water from 435 public systems [4]. Today, many of these systems are faced with an array of challenges, including excessive water loss and water quality problems. The latter includes the need to maintain an adequate disinfection residual while minimizing the creation of disinfection byproducts (DBPs). Such challenges have become especially acute with many of the small and rural utilities that make up most utilities in the Appalachian region of the United States [5].

According to the American Society of Civil Engineers (ASCE), water utilities in the country lose a staggering 22.7 million cubic meters of water daily, equivalent to over 9000 Olympic-sized swimming pools. This translates to an alarming 7.95 billion cubic meters of non-revenue water loss each year [6]. In 2019 alone, the United States lost an estimated $7.6 billion worth of treated water due to leaks [7]. In addition to lost revenue, such leaks have a significant carbon footprint and provide potential pathways for contaminants (including organic compounds) to intrude into the distribution system. Such contaminants may increase the required disinfection levels (and costs) and contribute to the formation of additional DBPs.

A significant number of water distribution systems in Kentucky have excessive water loss (i.e., greater than 15%). Indeed, several systems have water loss values exceeding 30%, with some experiencing water loss rates as high as 70%. These problems tend to be disproportionately greater in the Appalachia region of eastern Kentucky, which is associated with more mountainous topography. Such high values are frequently attributed to poor construction methods, excessive water pressures, insufficient maintenance and repair, inadequate fiscal resources (frequently associated with inadequate water rates), an inadequate number of trained operators, and poor management [8]. Many of these same systems have historically had problems with low chlorine (Cl) residuals and high DBP values [9]. Although not initially readily apparent, and if not adequately planned, attempts to address water loss in such communities can have an unanticipated negative impact on the associated water quality of the same systems.

This paper therefore demonstrates that an uncoordinated reduction in water loss can lead to an increase in trihalomethane concentrations along with a decrease in chlorine residuals to the extent that water quality standards are violated, using the Martin County water system in Kentucky, USA. This perhaps counterintuitive expectation is based on the premise that reducing water loss can increase water residence times (water ages) throughout the distribution network, providing more time for chlorine residual decay and the enhanced formation of DBPs, particularly trihalomethanes. To test this hypothesis, calibrated and validated hydraulic and water quality models of the Martin County system were used to evaluate the impact of reducing water loss rates in Martin County from 70% to an industry standard of 15% while keeping the original system operations unchanged. Thus, this study quantitatively shows how water loss reduction can paradoxically create water quality compliance challenges in rural, high-water-loss systems, and provides a framework for integrated planning that other small utilities can adapt.

## 2. Description of the Martin County System

**Figure 1.**
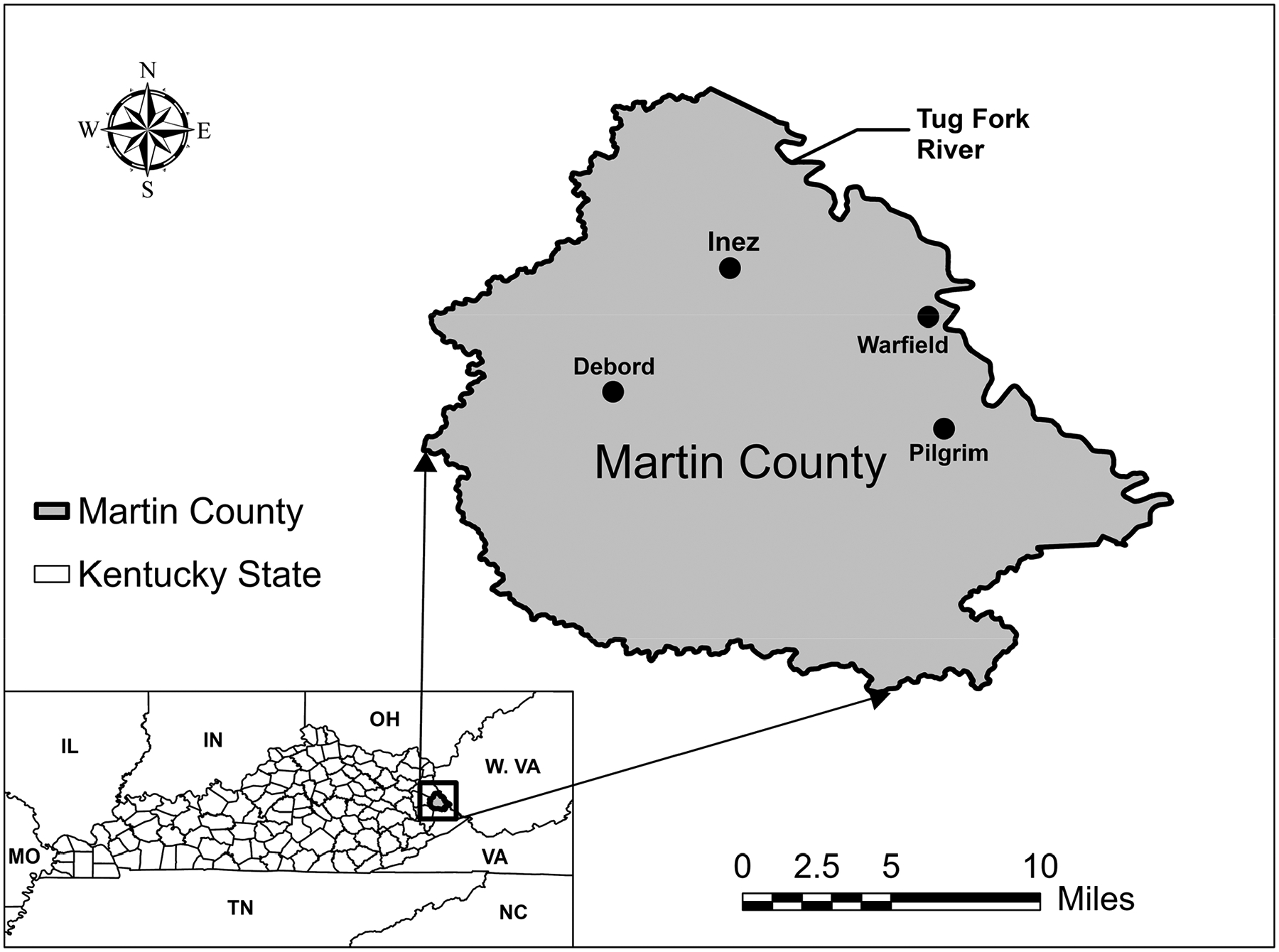
Location of Martin County, Kentucky.

Martin County is in the Appalachian region of Eastern Kentucky, near the border with West Virginia, and is considered one of the most economically distressed counties in the United States [10]. The county’s historical dependence on coal mining has left it vulnerable to the associated economic decline and infrastructure deterioration as the industry has contracted over the past few years. Despite these challenges, residents in Martin County face some of the highest water and sewer rates in the state of Kentucky. In 2024, the average monthly customer bill for potable water was approximately 18.9 m^3^ was estimated at $157.93 [11]. For many households, this cost is unaffordable, leading to reduced water use and increased reliance on alternative sources such as bottled water, springs, rainwater, or water from abandoned mines. In some cases, water theft has also been reported. These conditions place further strain on the already fragile distribution system and contribute to ongoing concerns about water quality and reliability. The situation has drawn national attention, with coverage by The Washington Post, The Los Angeles Times, and CNN [12–14]. Although Martin County’s case is extreme, it reflects broader issues faced by many rural communities in Appalachia, where limited funding, difficult terrain, and aging infrastructure present major barriers to safe and reliable water delivery [15]. Figure 1 shows the location of Martin County within the state of Kentucky and its proximity to the adjacent state of West Virginia.

The Martin County water system was originally constructed in 1968 for approximately 600 customers, largely living in the small town of Inez, KY, which is the county seat. Since then, the system has been consolidated with another water district also in Martin County and has continued to expand to serve other small communities as well as customers throughout the county. The system now serves approximately 3600 customers.

Irrespective of the small number of customers, the distribution system consists of approximately 366 km of pipe ranging in diameter from 76 to 254 mm. Most of the pipes are PVC; however, there appear to be some ductile iron pipes and asbestos cement pipes in the older parts of the system, mainly near the downtown area. Historically, the system has experienced water loss rates as high as 70% due to ongoing pipe breaks, largely attributable to poor construction methods and high system pressures (some exceeding 2070 kPa). The system also occasionally experiences boiling water advisories [16].

**Figure 2.**
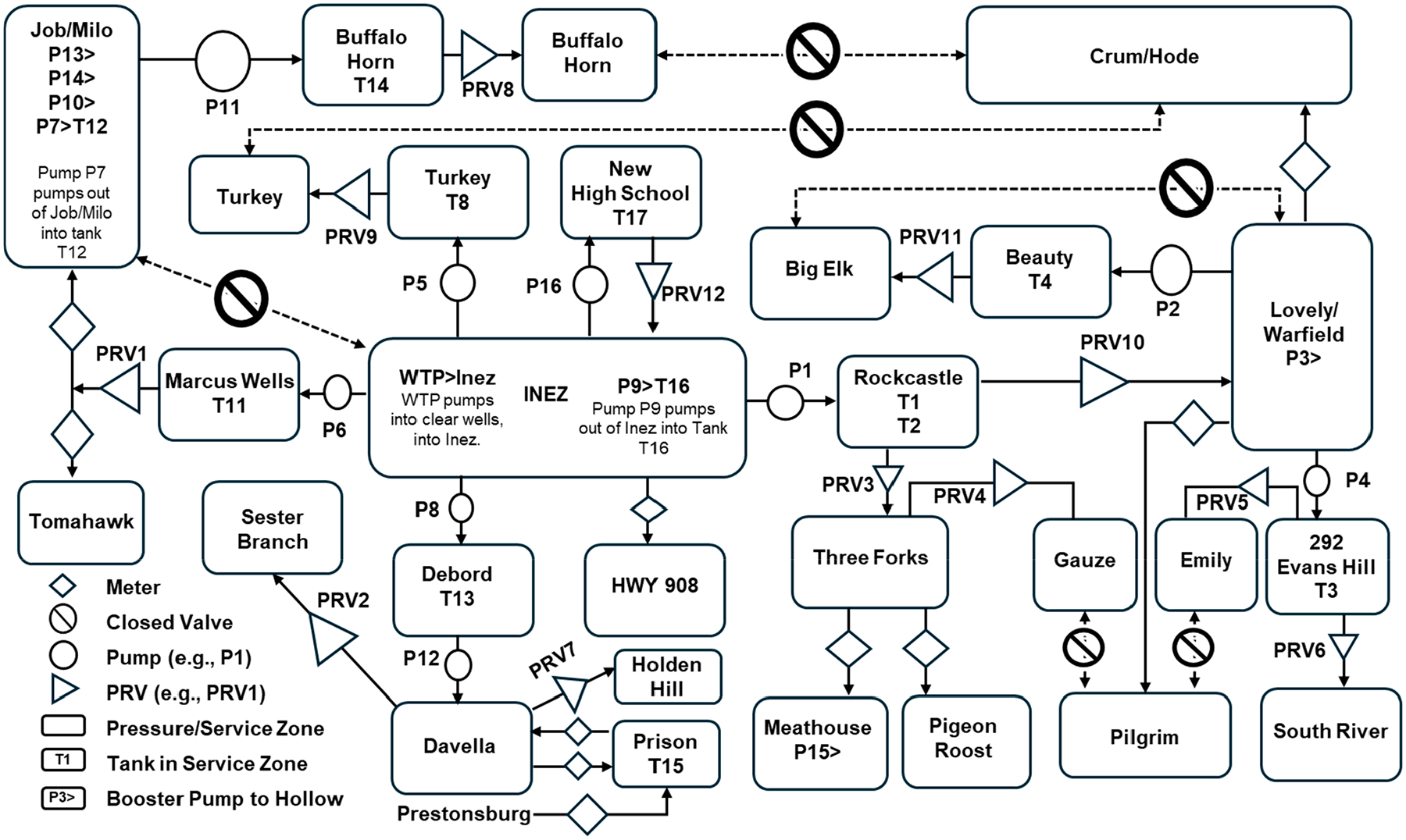
Schematic of the Martin County Water Distribution System.

Despite its size, the Martin County water distribution system is incredibly complex, as illustrated in Figure 2. Currently, the system is divided into 23 metered demand zones and contains 17 storage tanks or standpipes (only 14 of which were floating on the system) with a total capacity of nearly 11.4 million liters (ML), 17 pump stations, and 12 pressure-regulating valves. The complexity of the system is largely necessitated by the mountainous topography of the region and the tree-like structure of most of the distribution system.

**Figure 3.**
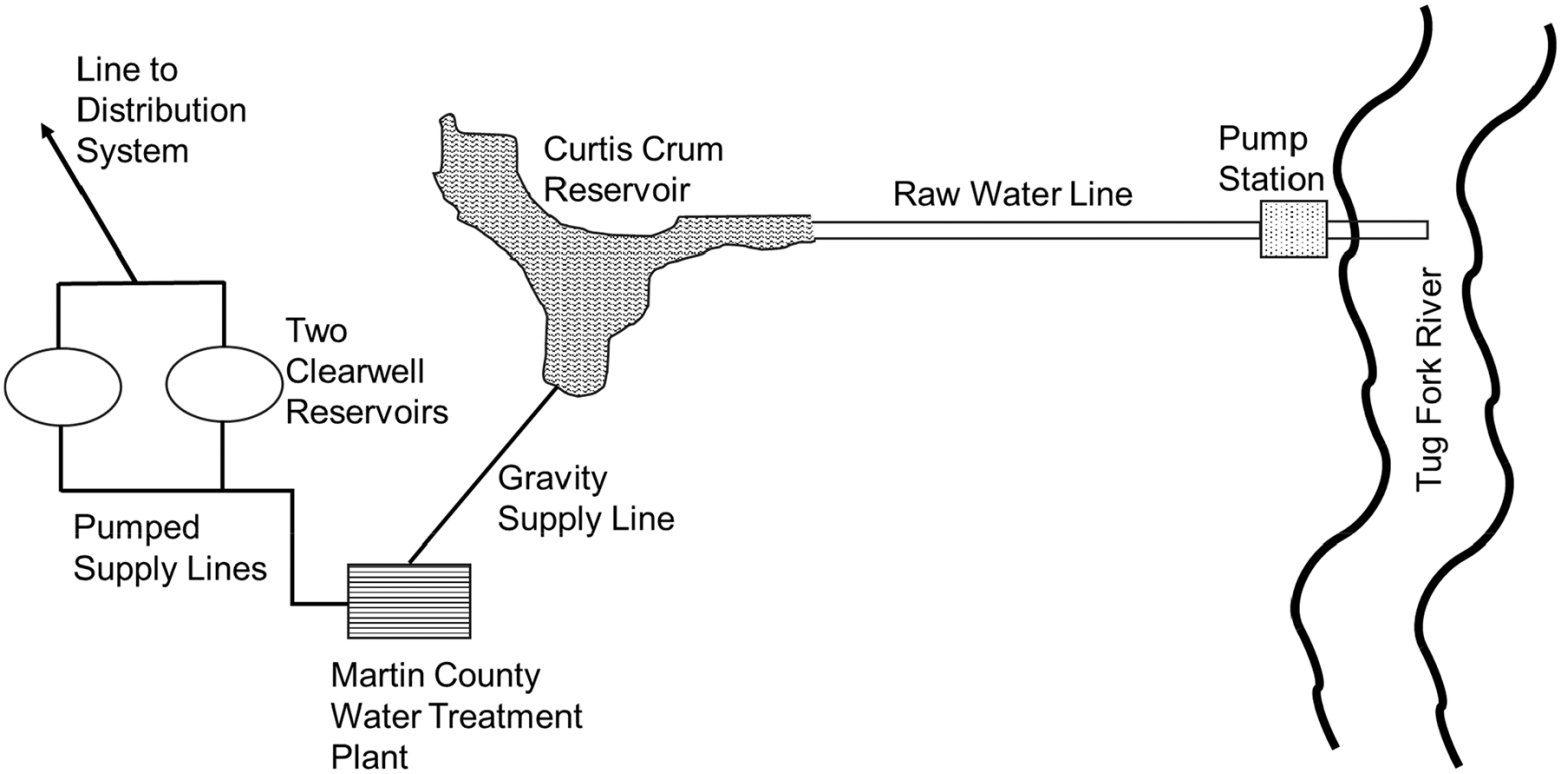
Schematic of the Martin County Water Supply and Treatment System.

Water for the county is currently provided from two sources: the Tug Fork River, which serves as the boundary between Kentucky and West Virginia, and a relatively small reservoir (the 6.5 ha Curtis Crum reservoir built in 1969), which is located between the river and Inez, just upstream of the water treatment plant. Figure 3 illustrates the water supply system and treatment system of Martin County. The 2.6 square kilometer Curtis Crum reservoir is currently undeveloped and can provide relatively good-quality water, although the reservoir has a limited capacity of approximately 370,000 cubic meters of storage. On the other hand, water from the river (which is normally inferior in quality) can be pumped to the headwaters of the reservoir, where it mixes with the water in the reservoir to provide water for the treatment plant. Operators consistently draw water from the reservoir, which can be replenished by pumping from the river.

The Martin County water treatment plant has a rated capacity of 7.6 ML/day and experiences an average daily demand of 5.3 ML/day. The water treatment plant consists of three circular clarifier/filter units, which are used for treating water. Once treated, the water is pumped into two 1.9 ML tanks located above the plant, which serve as the clear wells for the plant, before it is released by gravity into the distribution system (see Figure 3). Historically, raw water was treated with chlorine before the clarifier and then after filtration. This led to significant DBP violations up through 2017, when pre-chlorination was switched from before the clarifier to after the clarifier but before filtration. However, during summer periods, TTHM values leaving the plant have been observed to be as high as 0.04 mg/L, half the USEPA maximum contaminant level (MCL). This is typically associated with higher brominated TTHM species, attributed to higher bromide concentrations in the river, which is normally utilized during the summer and early fall months, due to an inadequate natural supply from the reservoir. Higher temperatures during the summer (i.e., >18 °C) have also been found to trigger a greater production of chloroform in the water [17]. Although the system currently meets compliance standards for disinfection byproduct (DBP) levels, ongoing research conducted by University of Kentucky scientists and citizen researchers in Martin County suggests that county-wide DBP concentrations may exceed what recent compliance sampling indicates [17,18].

## 3. Summary of the Model Development

**Figure 4.**
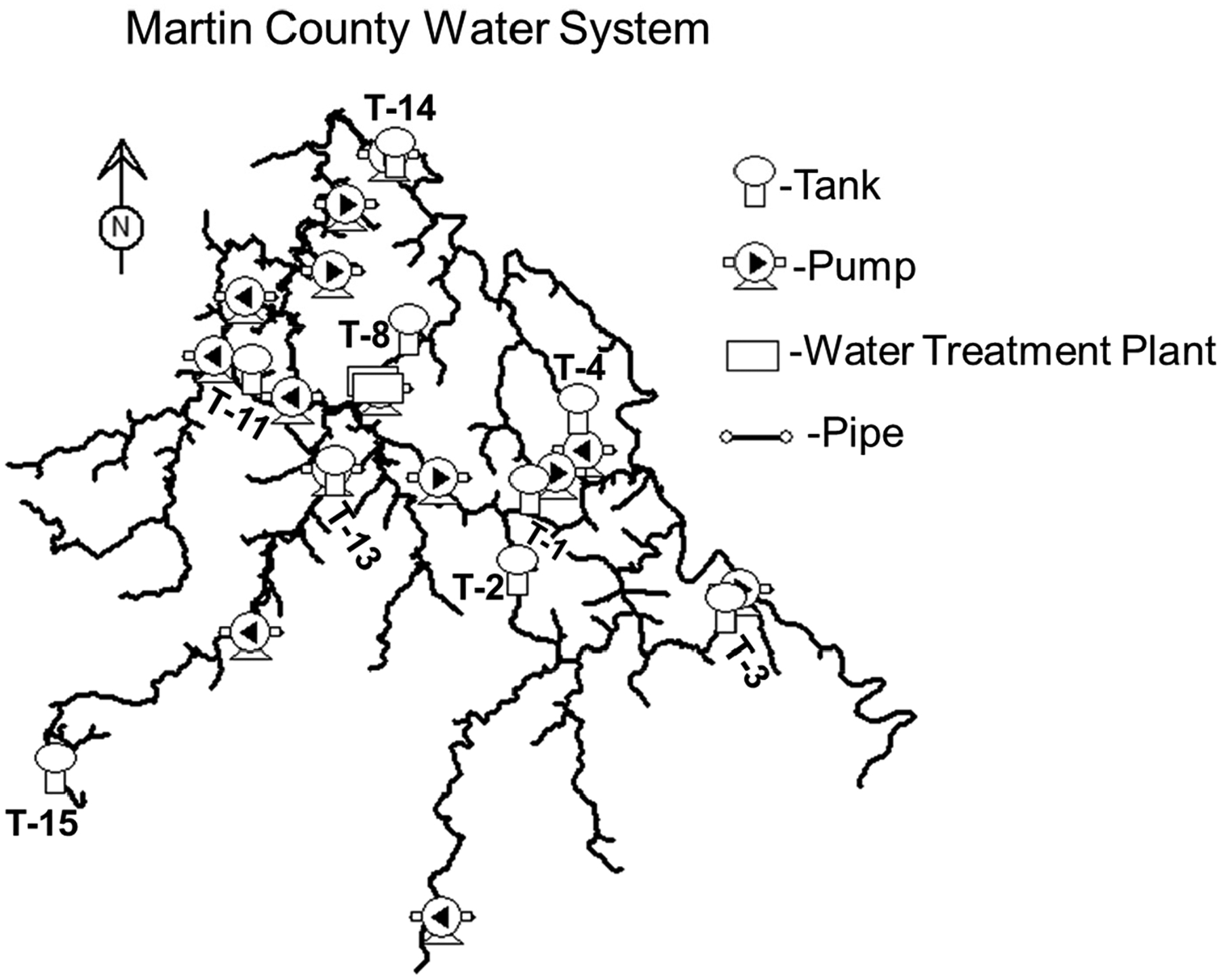
Map of the Martin County Water Distribution Network Showing Critical Tanks.

To test the hypothesis that water loss reduction can lead to an increase in trihalomethane concentrations and a decrease in chlorine residual values, an EPANET water quality model was developed and then applied to the Martin County water distribution system. The finalized model comprised 741 pipe segments and 673 junction nodes, together with 14 storage tanks, 17 pump stations, and 12 pressure-reducing valves (PRVs). A schematic representation of the system is provided in Figure 4.

**Figure 5.**
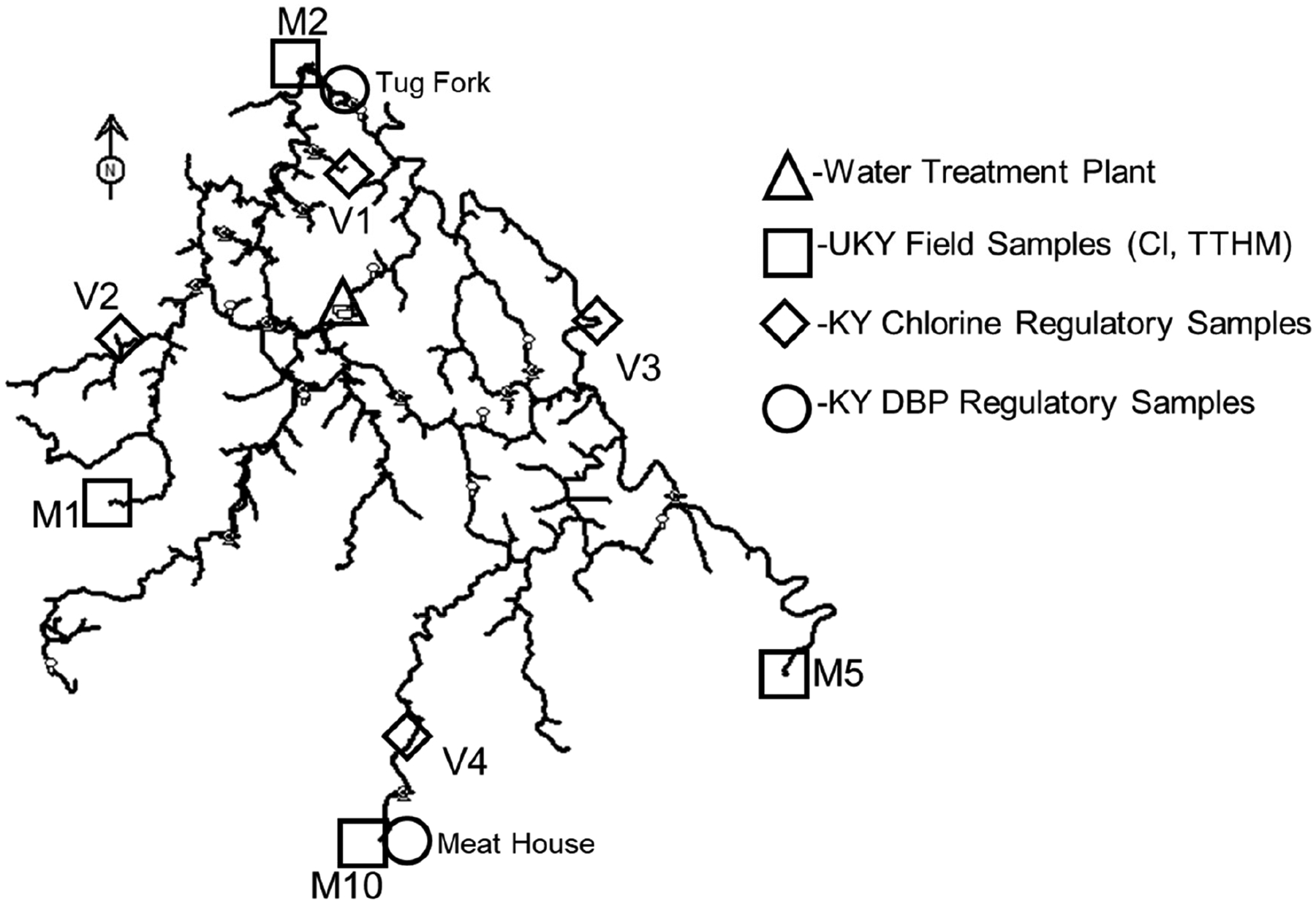
UKY field sites (□) used for model calibration, and regulatory chlorine (♢) and TTHM (○) sites used for validation in the Martin County Water System.

The model was applied for two different seasons (winter–spring and summer–fall), which were observed to yield different chlorine decay and trihalomethane growth characteristics. The application of the model required calibration of the hydraulic parameters (i.e., pipe diameters, pipe roughness, and nodal demands) and subsequent calibration of the water quality parameters (i.e., chlorine bulk decay coefficient, chlorine wall rate coefficient, and trihalomethane growth coefficient). This effort required multiple field campaigns to collect system operational and physical data, as well as repeated sampling trips to obtain water quality data at the treatment plant and throughout the distribution system. The monitoring locations used for model calibration and validation are shown in Figure 5. A detailed discussion of the model development process (including field sampling protocols, laboratory analyses, and quality assurance/quality control (QA/QC) protocols), along with the corresponding hydraulic and water quality calibration and validation results, is provided in Appendices A–C.

## 4. Modeling Water Loss Reduction Scenarios

To evaluate the impact of water loss reduction on distribution system water quality, a series of modeling scenarios was developed using the calibrated and validated hydraulic and water quality models (see Appendices A–C for detailed methodology and Figures A1–A9 and Tables A1–A6 for detailed results). The analysis was designed to test the hypothesis that reducing water loss could inadvertently affect water quality by increasing water residence times and promoting chlorine decay and DBP formation throughout the network.

Water loss reduction scenarios were modeled by reducing the total system-wide flow demands at each junction node. The baseline model (70% water loss) represents current conditions where total flow through the system includes both customer demand and water lost through leaks and/or theft. Two scenarios were evaluated: a moderate reduction to 30%, representing major pipe replacement and leak repair efforts, and a more aggressive reduction to 15%, consistent with best practices for system optimization. The 15% target reflects the performance benchmark recommended by AWWA [19] for cost-effective water loss control. When water loss is reduced, less total water flows through the system to serve the same customers, resulting in lower flow velocities throughout the distribution network. This translates to increased water residence times, thereby providing more time for chlorine decay and TTHM formation. This modeling approach assumes spatially uniform water loss reduction across the system.

For each scenario, the hydraulic model was executed to determine the resulting changes in flow velocities, water age, and hydraulic residence times throughout the network. The reduced water losses translated to lower demand at individual nodes, creating longer travel times from the treatment plant to end-users and increased stagnation in low-demand or peripheral areas of the system. The water quality implications of each water loss reduction scenario were evaluated using both chlorine residual and TTHM formation models. Chlorine residual concentrations were predicted at critical monitoring locations using the calibrated Kw values and updated demand inputs. Subsequently, the results were used to estimate TTHM concentrations with the TTHM models.

**Figure 6.**
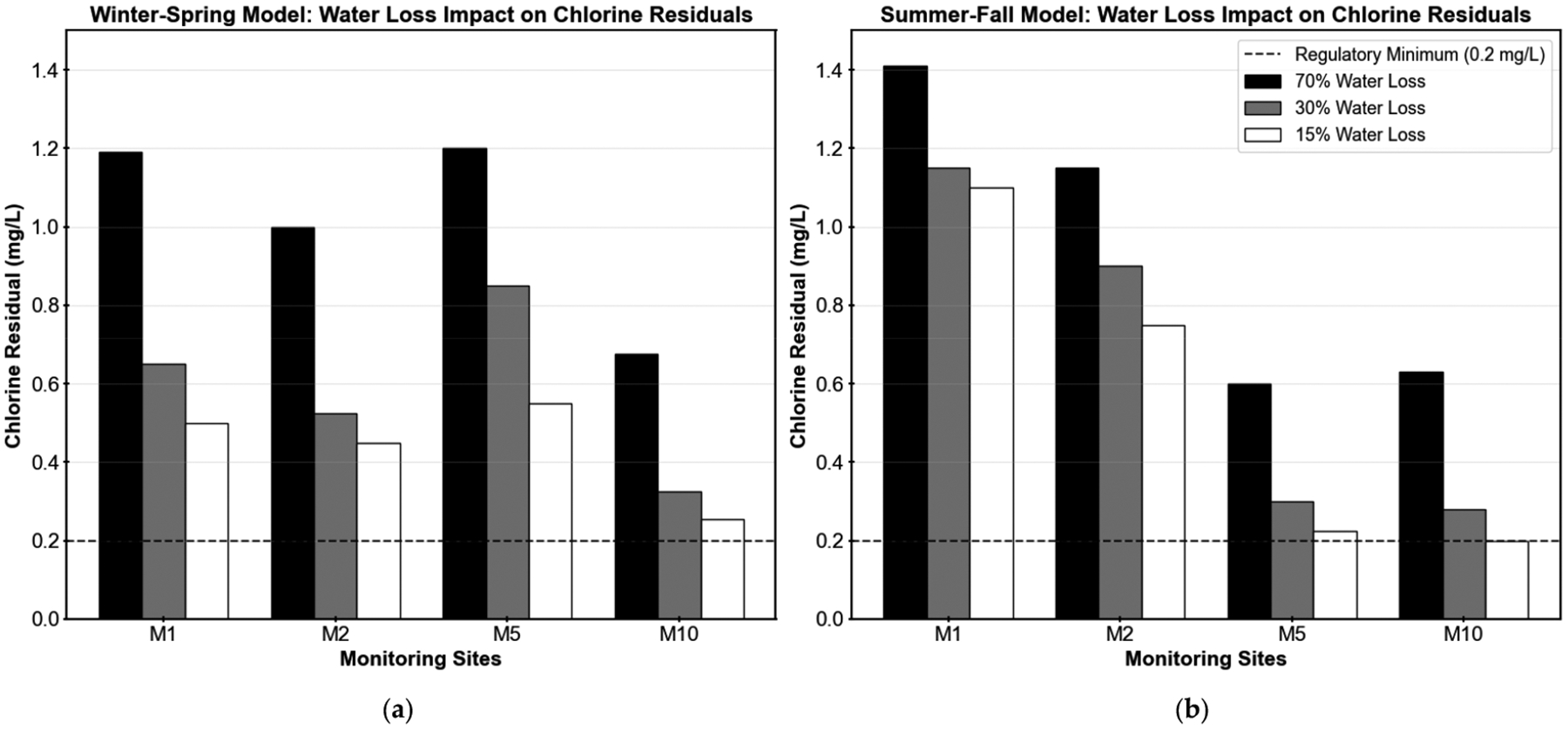
Effect of water loss reduction on chlorine residual concentrations at four monitoring locations (M1, M2, M5, and M10) in the Martin County water system, under three water loss scenarios (70%, 30%, and 15%). The horizontal dashed line indicates the USEPA minimum regulatory limit of 0.2 mg/L for chlorine residual. (**a**) Winter–spring model results; (**b**) summer–fall model results.

The analysis focused on the original calibration monitoring locations (M1, M2, M5, and M10), which are situated farthest from the treatment plant and are likely to be most affected by increased residence times (see Figure 5). The objective was to determine whether water loss reduction could lead to chlorine residuals falling below 0.2 mg/L or TTHM concentrations exceeding the regulatory limit of 0.080 mg/L. These locations serve as critical indicators of potential water quality trade-offs associated with improved hydraulic efficiency. Table 1 and Figure 6 illustrate the effects of water loss reduction on chlorine residual concentrations at the four monitoring sites under both winter–spring and summer–fall conditions. As water loss was reduced from 70% to 15%, a consistent decline in chlorine residuals was observed across all locations, due to longer residence times and less demand. In the winter–spring model, reductions in chlorine residuals ranged from 54% to 62%, with all sites maintaining chlorine levels above 0.2 mg/L. The summer–fall model showed more variable impacts, with reductions ranging from 22% to 68%. Sites M5 and M10 experienced the most significant decreases (63% and 68%, respectively), with M10 reaching the critical threshold of 0.2 mg/L under the 15% loss scenario. The differential seasonal response reflects the varying baseline chlorine concentrations and decay rates between the two models.

**Figure 7.**
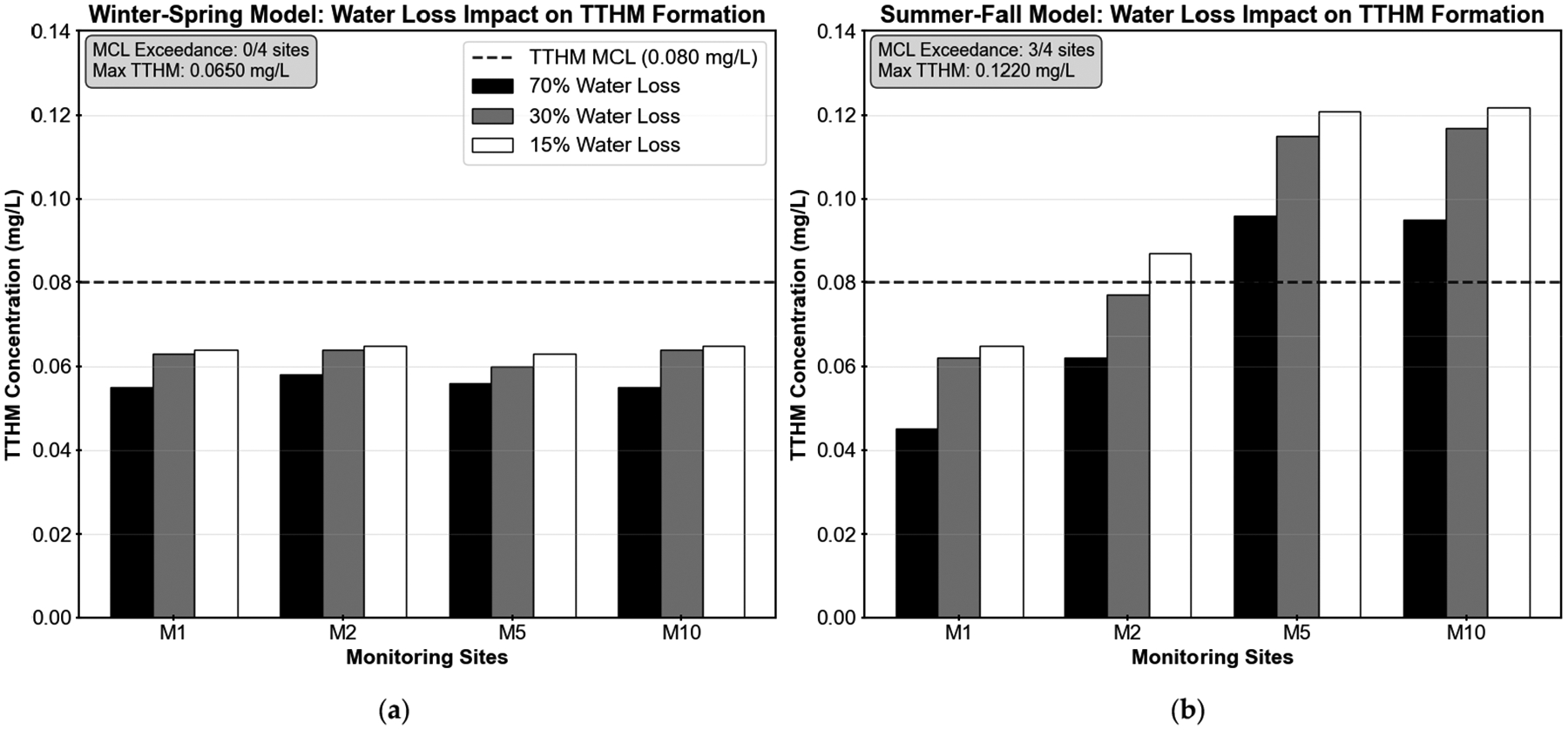
Effect of water loss reduction on total trihalomethane (TTHM) formation in the Martin County water distribution system. Three water loss scenarios were modeled: 70%, 30%, and 15% loss rates. The horizontal dashed line indicates the maximum contaminant level (MCL) of 0.080 mg/L for TTHMs. (**a**) Winter–spring conditions; (**b**) summer–fall conditions.

Correspondingly, Table 2 and Figure 7 present the predicted TTHM formation under the different water loss reduction scenarios. TTHM formation showed differential seasonal responses to water loss reduction. During winter–spring conditions, TTHM concentrations increased modestly by 12–18%, remaining well below the MCL of 0.080 mg/L with maximum predicted concentrations of 0.065 mg/L across all sites. Summer–fall conditions exhibited more pronounced increases of 26–44%, with sites M2, M5, and M10 exceeding the regulatory MCL under the 15% water loss scenario, reaching concentrations of 0.087 mg/L, 0.121 mg/L, and 0.122 mg/L, respectively. Site M1 remained below the MCL but showed significant increases from its baseline concentration. The higher TTHM formation observed during warmer months reflects the combined influence of higher temperatures on chlorine decay and the extended residence times caused by water loss reduction. These results demonstrate that, although reducing water loss improves system efficiency, it can also compromise water quality at locations with longer residence times or higher chlorine demand.

## 5. Discussion of Results and Possible Solutions

**Figure A1.**
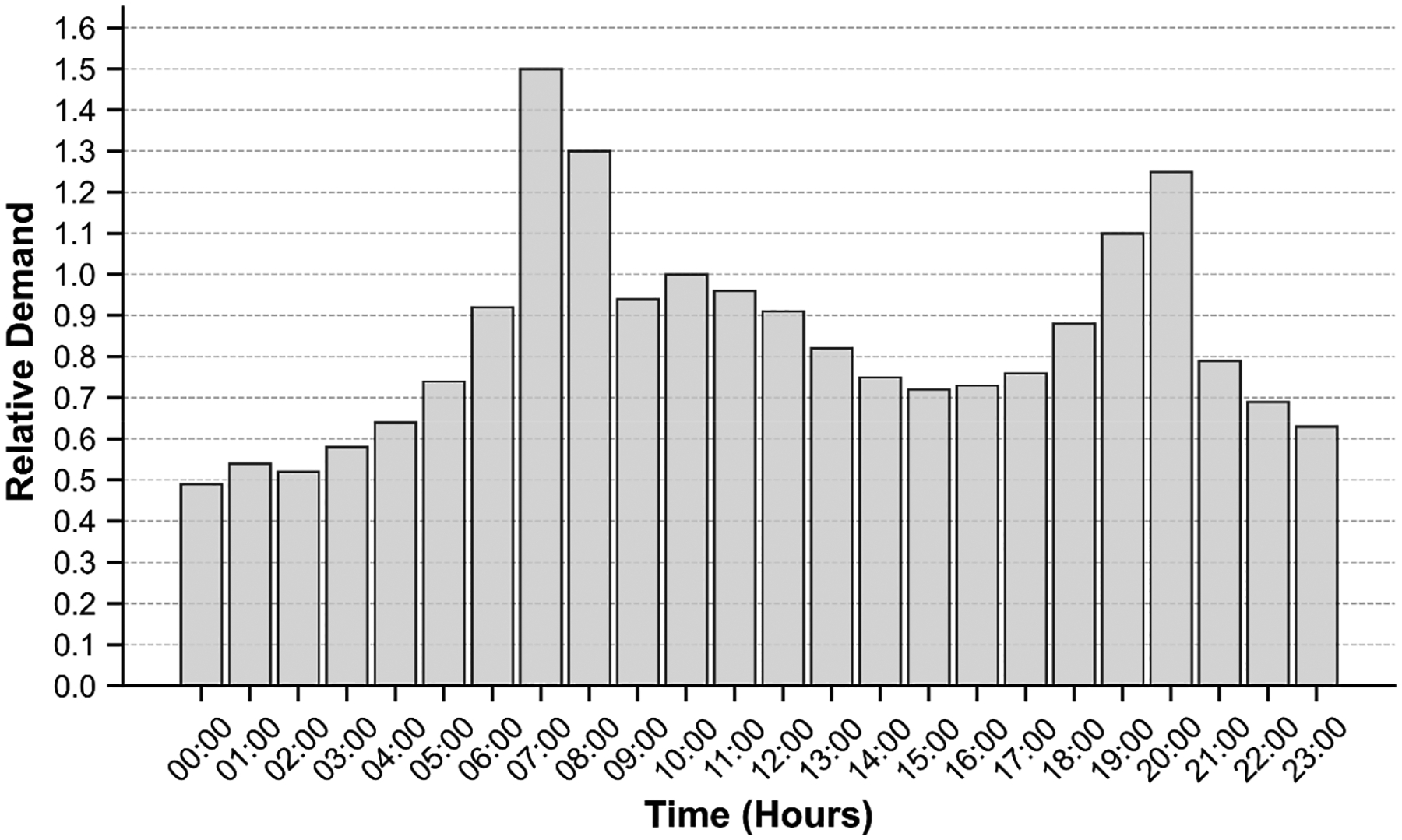
Representative Water Demand Pattern for Martin County Water System.

**Figure A6.**
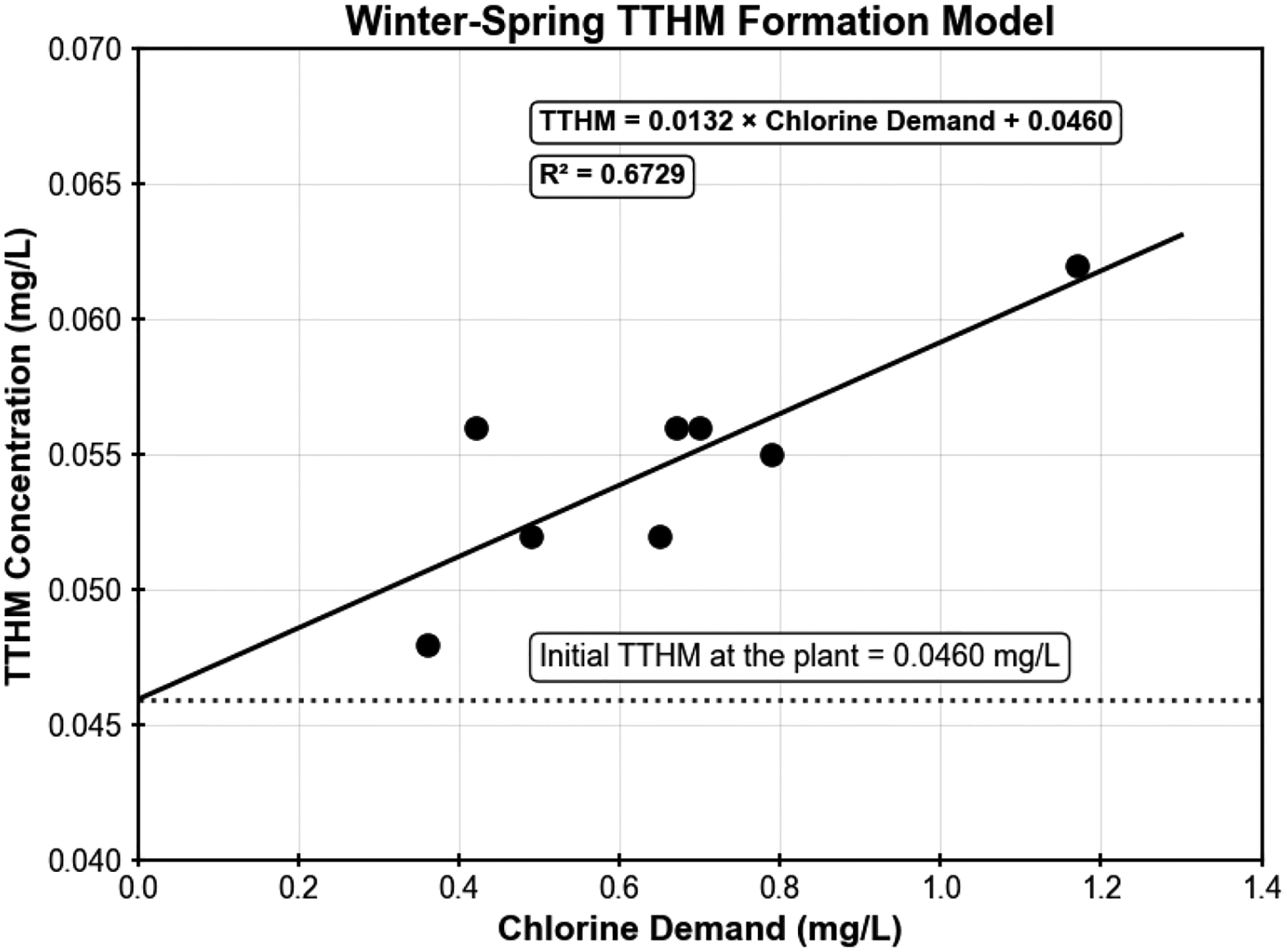
Winter–Spring model illustrating the correlation between chlorine demand and total trihalomethane (TTHM) formation.

**Figure A7.**
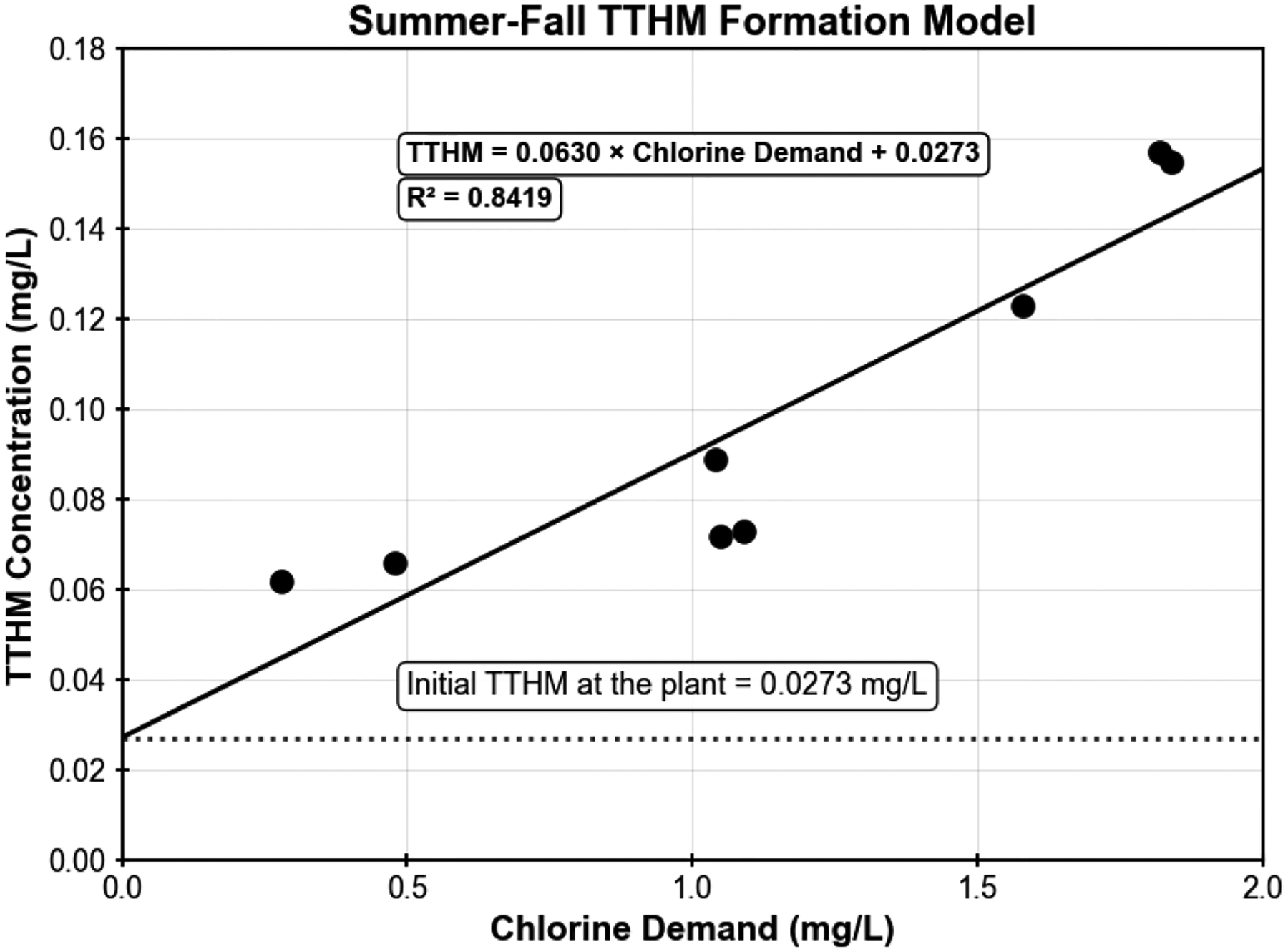
Summer–Fall model illustrating the correlation between chlorine demand and total trihalomethane (TTHM) formation.

**Figure A8.**
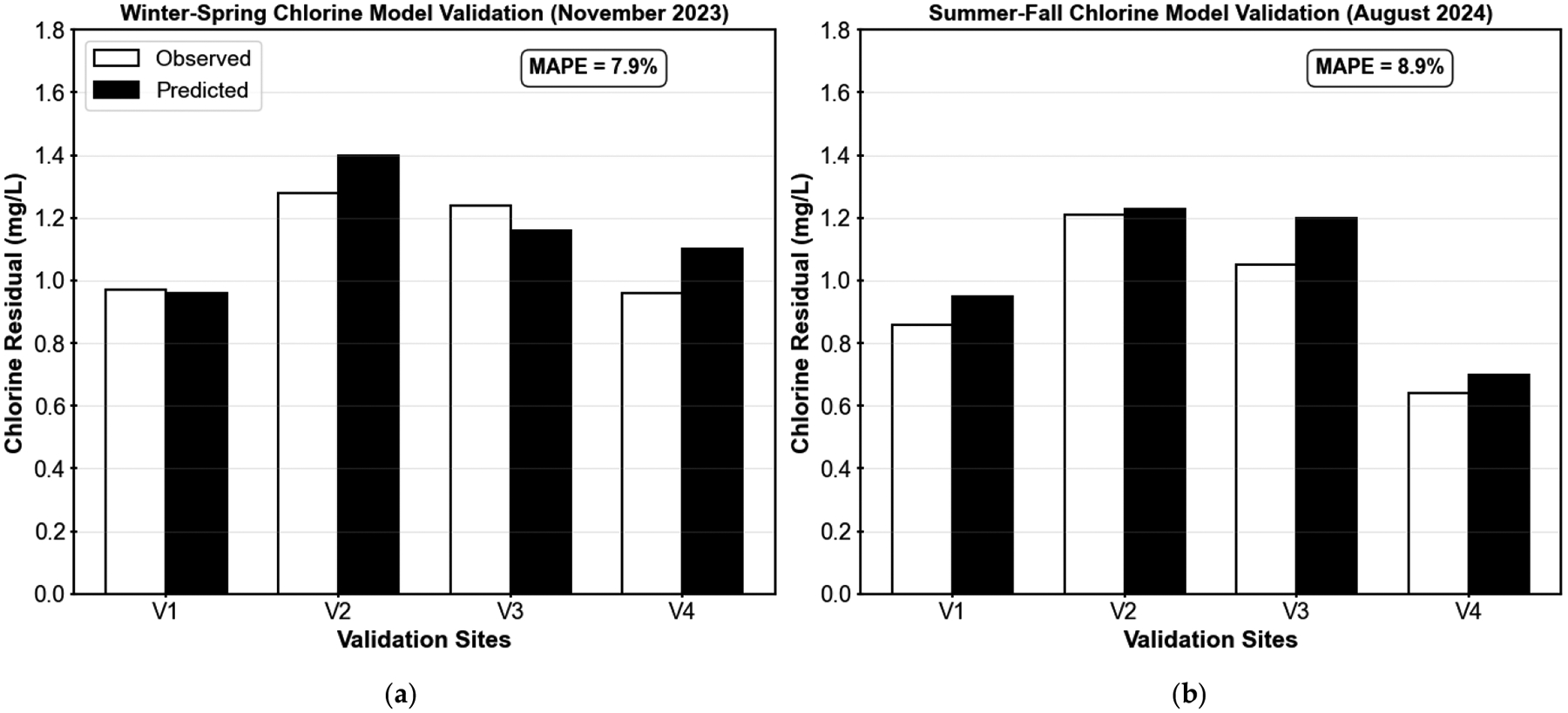
Comparison of observed and predicted chlorine residuals at four representative validation sites for the Martin County distribution system. (**a**) Results for the winter–spring model (November 2023) are compared across the four sites; (**b**) the results for the summer–fall model (August 2024) are also compared for each of the validation sites.

**Figure A9.**
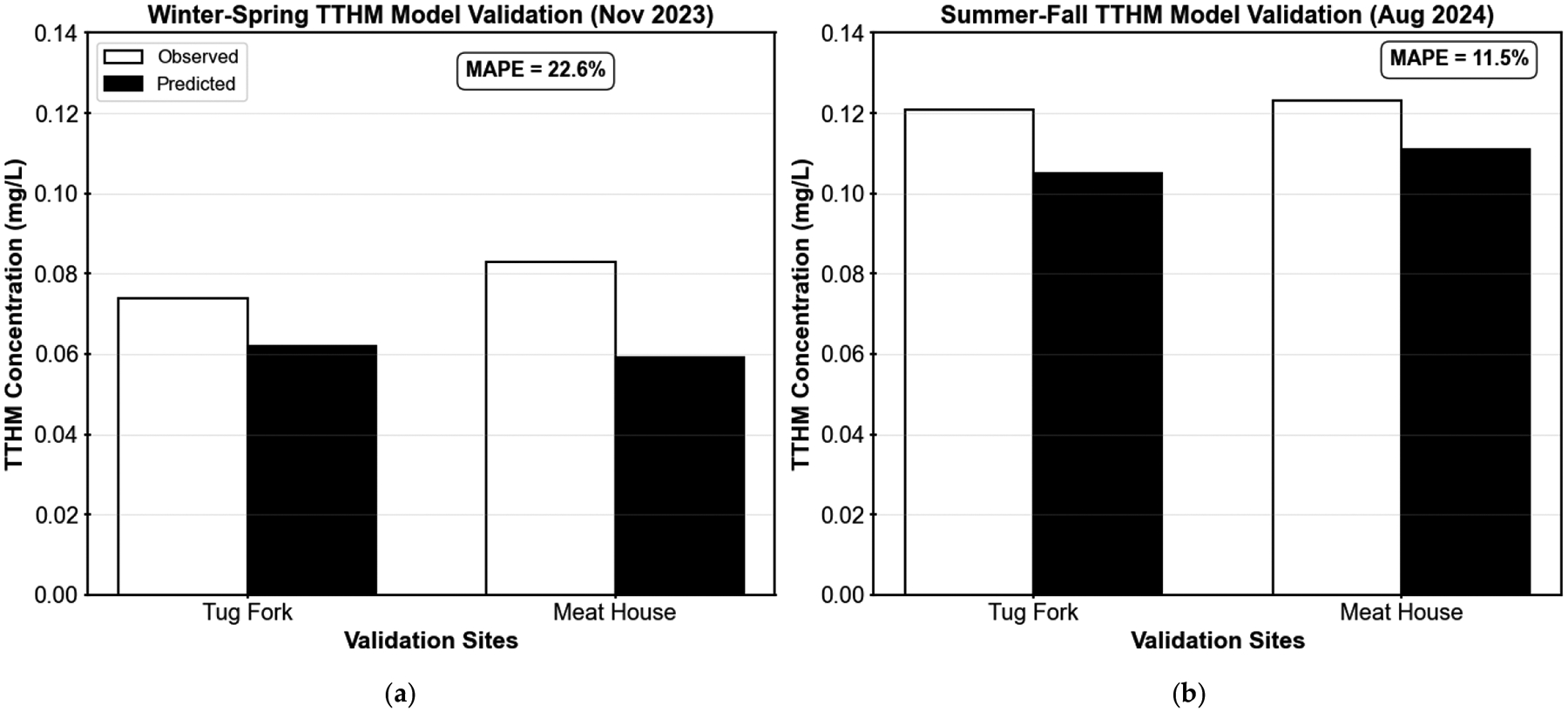
Comparison of observed and predicted total trihalomethane (TTHM) concentrations at Tug Fork and Meat House sites. (**a**) Results for the winter–spring model (November 2023) are compared at both validation sites; (**b**) The results for the summer–fall model (August 2024) are also compared for both validation sites.

The modeling results support this study’s hypothesis that reducing water loss can inadvertently deteriorate water quality within distribution systems. Decreasing water loss from 70% to the industry-recommended 15% benchmark [19] revealed a fundamental trade-off between hydraulic efficiency and water quality compliance, challenging the conventional assumption that all infrastructure improvements inherently benefit system performance. To ensure confidence in these findings, the hydraulic and water quality models were calibrated and validated (detailed results in Appendices A–C and Figures A1–A9 and Tables A1–A6). The models demonstrated satisfactory to good performance, with chlorine residual models achieving MAPE values of 2.8–5.4% during calibration and 7.9–8.9% during validation (Tables A3 and A5, and Figure A8, Appendix C). TTHM models showed greater seasonal variability, with calibration errors of 3.3% (winter–spring) and 23.9% (summer–fall), and validation errors of 22.6% (winter–spring) and 11.5% (summer–fall) against independent regulatory monitoring data (Tables A4 and A6, and Figure A9, Appendix C). The enhanced TTHM formation during warmer months reflects the temperature-dependent nature of chlorine decay and DBP formation reactions. Additionally, the summer–fall model exhibits a steeper chlorine demand-TTHM relationship (slope = 0.0630) compared to the winter–spring model (slope = 0.0132), indicating accelerated DBP formation kinetics at higher temperatures (Figures A6 and A7, Appendix B). All performance metrics fall within acceptable criteria (Table A1, Appendix A), confirming the models reliably capture the hydraulic-water quality relationships that govern the distribution system’s behavior.

The counterintuitive water loss–water quality tradeoff was particularly pronounced across seasonal conditions. Winter–spring scenarios showed consistent chlorine residual declines of 54–62% across all monitoring sites, with all locations maintaining adequate residuals above the 0.2 mg/L regulatory minimum despite notable reductions. Summer–fall conditions presented more variable impacts, with chlorine reductions ranging from 22 to 68%. Critically, site M10 reached the 0.2 mg/L threshold under the 15% water loss scenario, indicating potential compliance risks at peripheral system locations.

TTHM formation showed differential seasonal responses to water loss reduction. During winter–spring conditions, TTHM concentrations increased modestly by 12–18%, remaining well below the MCL of 0.080 mg/L with maximum predicted concentrations of 0.065 mg/L. Summer–fall conditions exhibited more pronounced increases of 26–44%, with sites M2, M5, and M10 exceeding the regulatory MCL under the 15% water loss scenario, reaching concentrations of 0.087 mg/L, 0.121 mg/L, and 0.122 mg/L, respectively (see Figure 7). These exceedances represent potential regulatory violations that would need mitigation measures.

These results are particularly relevant for rural Appalachian systems like Martin County, where high water loss, complex terrain, and limited funding constrain system management. Utilities facing similar challenges must recognize that aggressive water loss reduction, if not carefully planned, may exacerbate existing water quality issues. Thus, they must evaluate the sequence and scope of infrastructure improvements. Systems currently struggling with DBP compliance may need to address water quality issues before or concurrently with efforts to reduce water loss to avoid creating additional regulatory violations. To mitigate these impacts, integrated solutions are essential. Recommended strategies include installing automated booster chlorination stations at strategic locations to maintain adequate residuals during extended residence times, phasing leak repairs starting with high-flow areas, and adapting seasonal operations, such as chlorine dosing, optimized tank management, and flushing schedules, to minimize stagnation and DBP formation. Real-time monitoring through SCADA systems can enable dynamic response to changing system conditions, while establishing district metered areas (DMAs) can help isolate and manage pressure and flow impacts at a localized scale. Importantly, utilities should align water loss and water quality improvements through integrated planning and funding mechanisms and work proactively with regulators to develop compliance timelines that reflect the interconnected nature of these challenges. This study highlights the importance of holistic infrastructure management in rural water systems, where addressing one issue can inadvertently create another without careful coordination.

## 6. Study Limitations and Future Research Directions

The results of this research offer important insights into the relationships between water loss and water quality; however, a few limitations should be noted. This analysis focused on a single utility with unique operational and topographic characteristics, which may limit the generalizability of specific quantitative results, though the underlying relationships and modeling approaches should be broadly applicable to similar rural systems. This study also concentrated on TTHMs, without consideration of HAAs or emerging DBPs, such as haloacetonitriles, ketones, nitrosamines, or iodinated compounds, which were beyond the scope and funding of the study. Additionally, while the modeling incorporated extended-period simulations to capture daily operational variability, it did not explore broader dynamic scenarios such as emergency events, extreme weather conditions, or dynamic chlorine dosing.

Furthermore, several additional questions were identified but were beyond the scope of this study. While many of these questions represent potential limitations of the present work, they also provide fertile ground for future research.

1. In the current study, water loss reduction was modeled through a system-wide reduction in water demand at each node. Future research should investigate the impact of non-uniform reductions (e.g., by demand zone), which may inform the utility about which demand zones to prioritize for water loss reduction.
2. Future research should explore the explicit impact of the raw water source (i.e., Crum Reservoir or the Tug Fork River) on the production of TTHMs. This may guide the utility when different sources of raw water might be preferable for the reduction in TTHM formation. A significant reduction in water loss might allow for the exclusive use of the reservoir, thereby allowing for lower TTHM values.
3. Future modeling should explore the potential impact of an increase in customer demand (thereby decreasing travel times) in response to a potential decrease in water rates caused by a reduction in water loss.
4. Future modeling should explore the potential impact of different tank operation strategies on TTHM concentrations. Currently, most of the tanks in Martin County are kept as full as possible to provide adequate emergency water in case of a major line break. With a reduction in water loss, it may be possible to operate the tanks to provide better tank turnover, thereby reducing the residence time in the tanks and improving the water quality.
5. Future modeling should explore the use of more strategic main flushing afforded by a decrease in water loss. Short-term benefits could be associated with evacuating older water with possibly higher DBP values, and long-term benefits could be associated with scouring out biofilms and other materials on the pipe walls that might be exerting a stronger chlorine demand, thereby reducing the overall chlorine levels required to maintain an adequate chlorine residual across the system.
6. Future modeling should explore the applicability of the results of this study and the above additional modeling to other rural systems in Appalachia to confirm the general applicability of these results to other systems.
7. Future research should include economic analysis comparing the costs and benefits of water loss reduction versus water quality mitigation strategies (e.g., booster chlorination, enhanced monitoring, operational adjustments). Such an analysis would provide utilities with a more complete decision-making framework for prioritizing infrastructure investments and operational improvements.

Results from such future modeling are expected to provide useful guidance to utility management as it relates to (1) the prioritization of water loss areas, (2) the prioritization of raw water sources, (3) the setting of water rates, (4) tank operations, and (5) main flushing. Such issues provide a roadmap for future research in this area and the potential for improved management and operation of such rural water systems.

## 7. Conclusions

This study demonstrated the complex tradeoff between water loss reduction and trihalomethane concentrations in rural distribution systems, using Martin County, Kentucky, as a representative case. Coupled hydraulic and water quality modeling showed that reducing water loss from 70% to 15% significantly increased water residence times, resulting in chlorine residual declines of 22–68% (with one site reaching the critical 0.2 mg/L threshold) and TTHM increases of 12–44%, with more pronounced impacts during summer–fall conditions. These outcomes confirm that aggressive loss reduction, while improving hydraulic efficiency, can lead to regulatory violations and degraded water quality if not managed carefully. The models achieved satisfactory to good performance across both calibration and validation, with performance metrics meeting established criteria despite expected seasonal variability, supporting confidence in the modeling approach and its applicability to similar systems in Appalachia and beyond.

In summary, and despite those identified study limitations, we feel confident in recommending that utility managers adopt integrated planning strategies that better align water loss control with water quality goals. Regulators should support flexible compliance frameworks and capacity building for small systems. Policymakers and funding agencies should promote integrated investment approaches and enable technology transfer from larger systems to small rural utilities. Managing rural water systems requires a holistic approach that balances efficiency, compliance, and affordability.

## Figures and Tables

**Table 1.** Impact of Water Loss Reduction on Chlorine Residuals.

Model	Site	70% Loss (mg/L)	30% Loss (mg/L)	15% Loss (mg/L)	Percent Decrease (70%→15%)
Winter–Spring	M1	1.190	0.650	0.500	58%
	M2	1.000	0.525	0.450	55%
	M5	1.200	0.850	0.550	54%
	M10	0.675	0.325	0.255	62%
Summer–Fall	M1	1.410	1.150	1.100	22%
	M2	1.150	0.900	0.750	35%
	M5	0.600	0.300	0.225	63%
	M10	0.630	0.280	0.200	68%

**Table 2.** Impact of Water Loss Reduction on total trihalomethane (TTHM) Formation.

Model	Site	70% Loss (mg/L)	30% Loss (mg/L)	15% Loss (mg/L)	Percent Increase (70%→15%)
Winter–Spring	M1	0.055	0.063	0.064	16%
	M2	0.058	0.064	0.065	12%
	M5	0.056	0.060	0.063	13%
	M10	0.055	0.064	0.065	18%
Summer–Fall	M1	0.045	0.062	0.065	44%
	M2	0.062	0.077	0.087	40%
	M5	0.096	0.115	0.121	26%
	M10	0.095	0.117	0.122	28%

**Figure A2.**
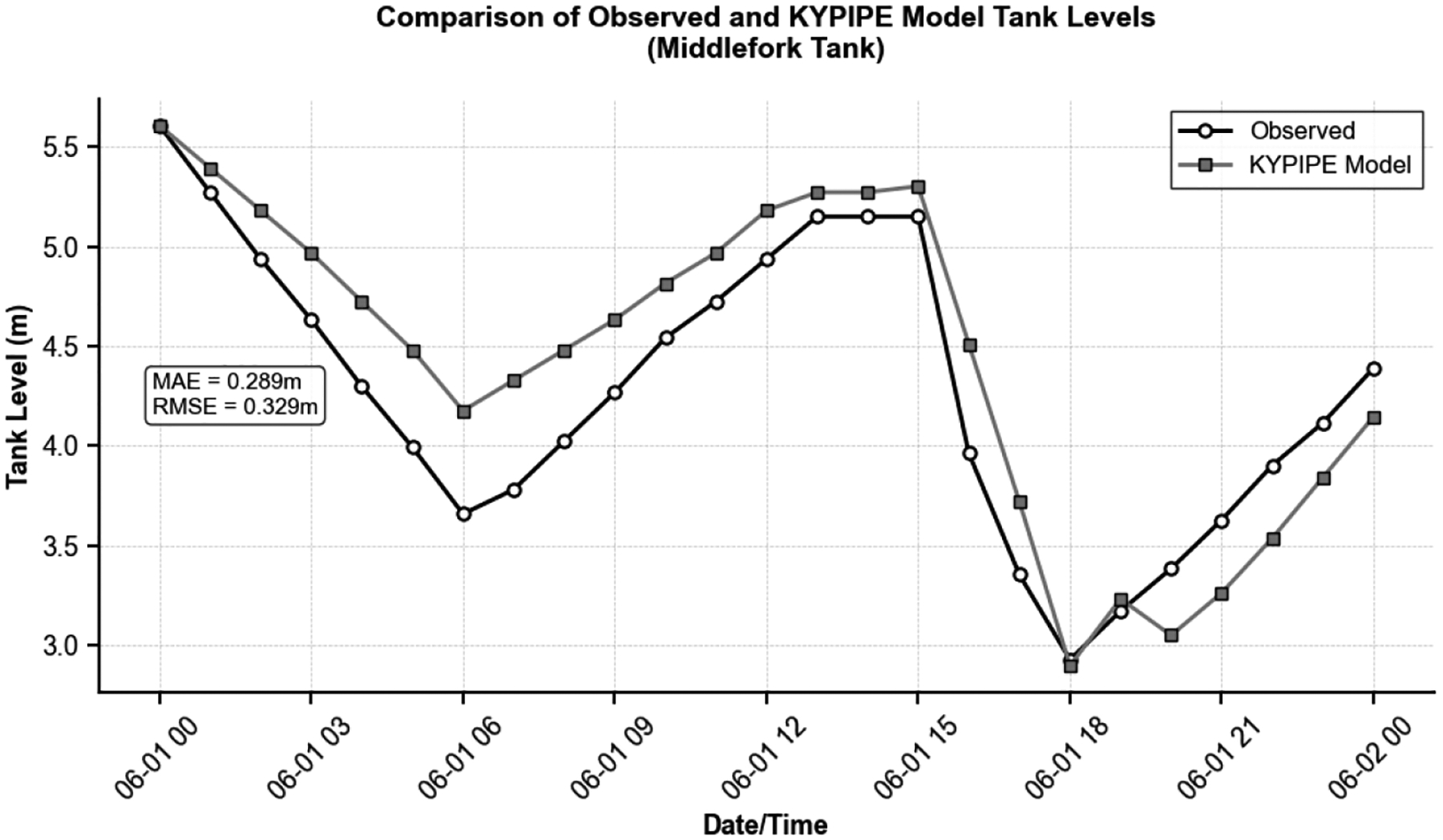
Comparison of observed and simulated tank levels for Tank-13 (Middlefork Tank).

**Figure A3.**
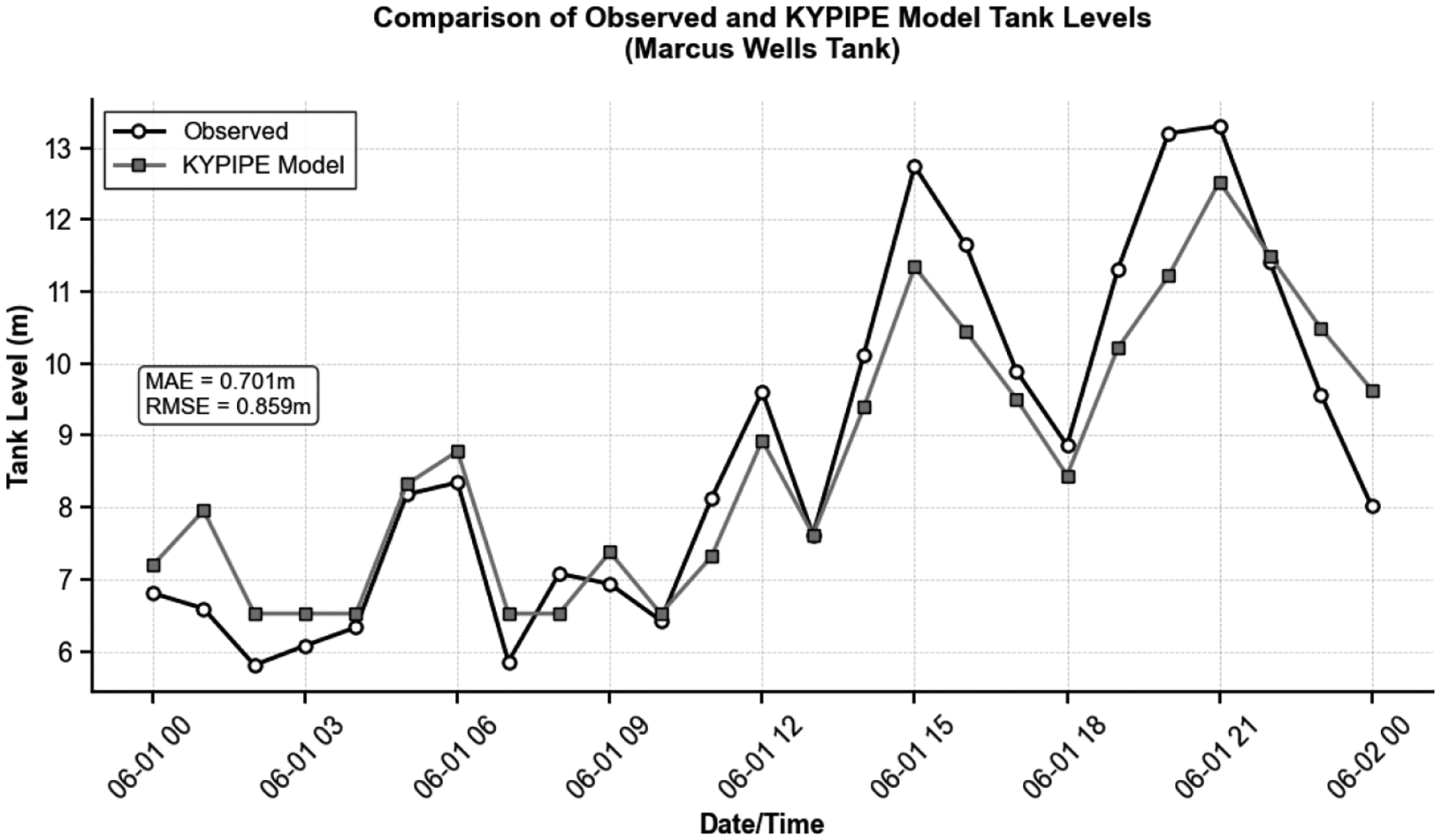
Comparison of observed and simulated tank levels for Tank-11 (Marcus Wells Tank).

**Figure A4.**
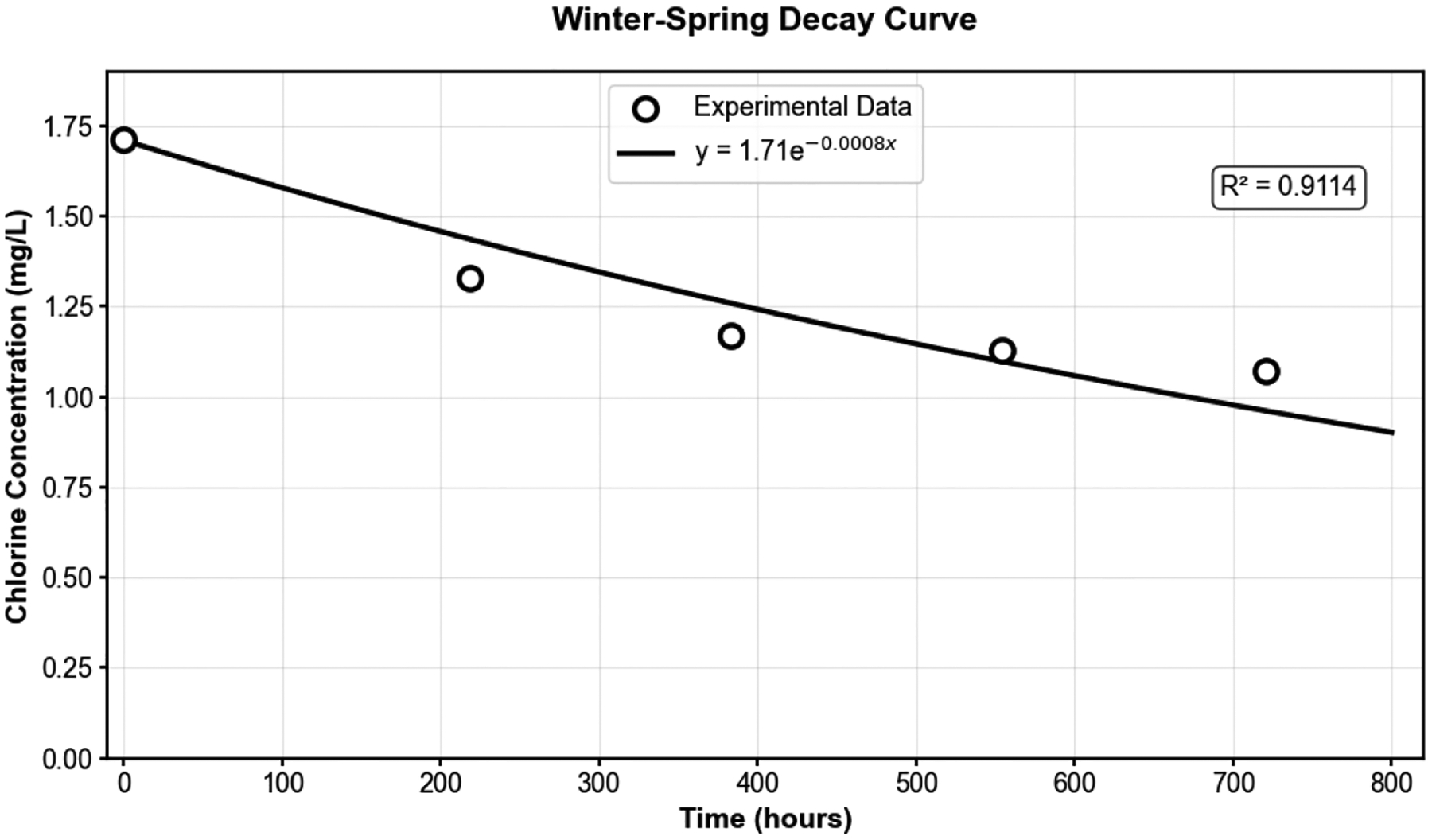
Winter–Spring Model Decay Curve for the Martin County System.

**Figure A5.**
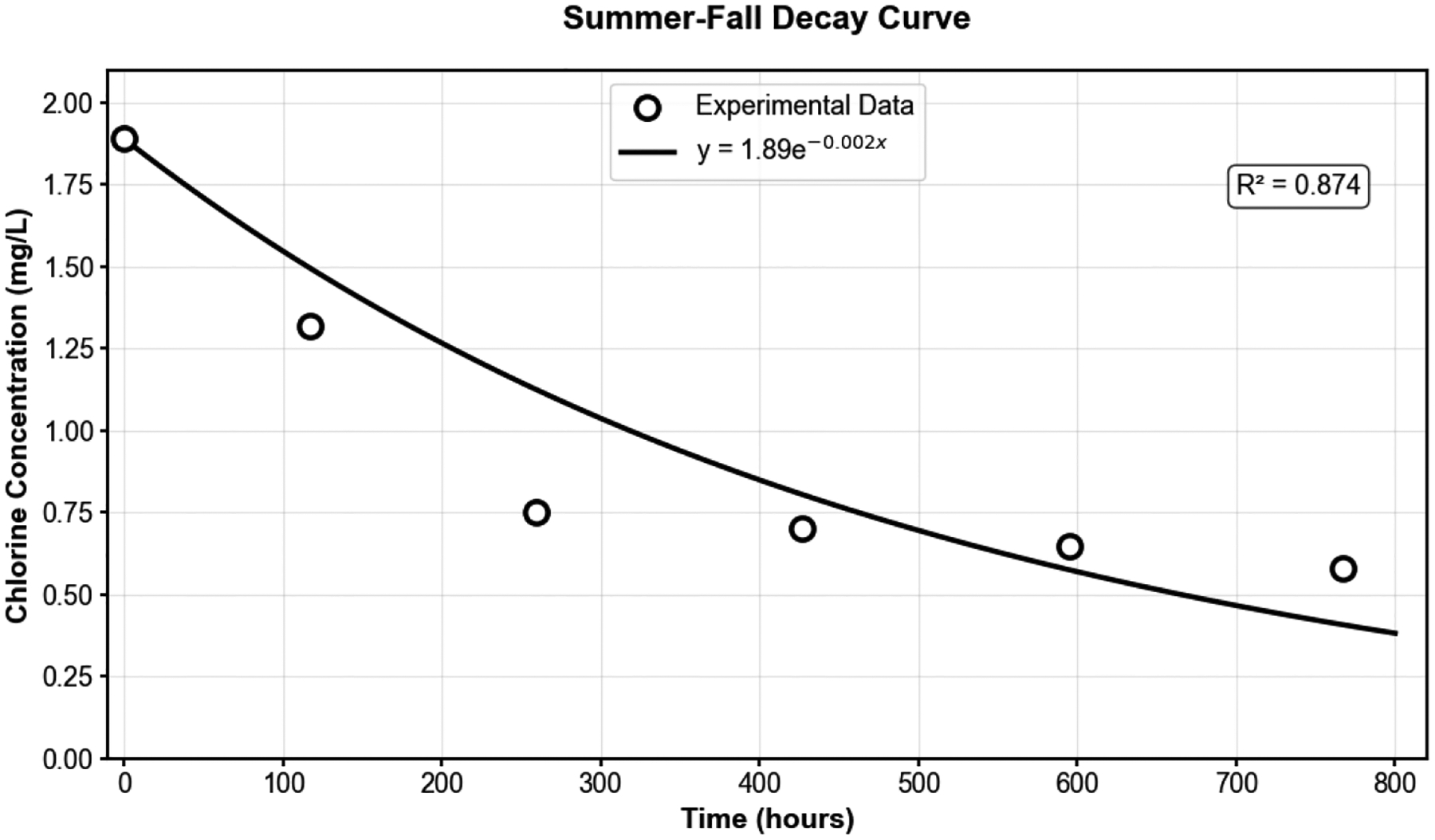
Summer–Fall Model Decay Curve for the Martin County System.

## Data Availability

Field measurement data supporting the water quality modeling results are available from the corresponding author upon reasonable request. Restrictions apply to the availability of the hydraulic model files and detailed infrastructure data; data were obtained from the Martin County Water District and are available from the authors with the permission of the Martin County Water District. Publicly accessible network information is available through the Kentucky Infrastructure Authority, accessible through their Water Resources Infrastructure System (WRIS) (https://kia.ky.gov/WRIS/Pages/WRIS-Portal.aspx, accessed on 9 October 2025).

## Appendix A. Development of the Hydraulic Model

The first step in testing our hypothesis was to develop a hydraulic model of the Martin County water distribution system. This process was facilitated through collaboration with the system’s operational staff and their engineering consultant; however, it was complicated by multiple management transitions during our five-year study period. Initially, the utility was managed by local personnel overseen by a utility board appointed by the county judge executive. The Kentucky Department of Energy and Environment subsequently intervened and mandated that the county contract with an external management company (Alliance Water) to operate the utility. After working for three years with the initial Alliance Water leadership team, senior management was replaced, necessitating the reestablishment of working relationships with the new leadership.

At the project’s outset, the local utility authorized our research team to obtain a rudimentary computer model previously created in KYPIPE from their engineering consultant. We integrated this model with spatial GIS files to provide geo-referenced background mapping and component elevations. The network topology and features (including pipe materials and diameters) were then cross-referenced against an online map developed by the Kentucky Infrastructure Authority, accessible through their Water Resources Infrastructure System (WRIS) (https://kia.ky.gov/WRIS/Pages/WRIS-Portal.aspx, accessed on 9 October 2025). Discrepancies between the two datasets were reconciled through meetings with utility personnel, though some data ambiguities persisted due to incomplete historical records. When Alliance Water assumed management, we gained access to their internal GIS dataset, which revealed additional inconsistencies. Through this comprehensive data reconciliation process, involving the synthesis of multiple datasets and extensive collaboration with utility personnel and their consultants, Gautam [20] finalized a foundational model that served as the starting point for this study. Additional details regarding pumps and storage tanks were verified using as-built drawings and contract documents housed in the physical map room at the Martin County water treatment plant. This information was used to support subsequent model calibration and water quality analysis. The resulting model distribution network is shown in Figure 4.

### Appendix A.1. Model Performance Evaluation Metrics

Model performance was evaluated using several statistical metrics widely used in hydraulic and water quality modeling studies to assess the agreement between observed and predicted values. These included the Root Mean Square Error (RMSE), the Mean Absolute Error (MAE), the Mean Absolute Percentage Error (MAPE), and the Coefficient of Determination (R^2^). The American Water Works Association (AWWA) [21] and other modeling references [22,23] emphasize that calibration adequacy must be evaluated in the context of a model’s purpose, the quality of the data, and system-specific conditions, rather than rigid universal thresholds. However, general performance criteria from the literature, shown in Table A1, provide useful context for interpreting the results.

**Table A1.** Statistical metrics and performance criteria for model evaluation.

Metric	Acceptable Criteria	References
MAE	<0.5 × standard deviation of observed values	[24–26]
RMSE	<0.5 × standard deviation of observed values	[26,27]
MAPE	<25% generally acceptable	[26,28]
R^2^	>0.6 generally acceptable	[24,26]

In this study, MAE and RMSE were used to evaluate hydraulic model calibration (tank levels). At the same time, MAE, MAPE, and R^2^ were applied to water quality model performance (chlorine residuals and TTHM predictions). Model performance was interpreted relative to the target criteria and the specific operational context of the Martin County system.

### Appendix A.2. Hydraulic Model Calibration Procedures

Initial estimates of Hazen-Williams roughness coefficients (C-factors) were obtained by conducting field tests at seven sites across the distribution network following AWWA standard methods [21,29,30]. Tests were performed on pipes of three different materials (i.e., PVC, asbestos cement (AC), and ductile iron) with diameters of approximately 100, 150, and 200 mm. Measured C-factors ranged from a low of 94 for AC pipe to a high of 145 for PVC pipe. Since the system is predominantly composed of PVC piping, an average C-factor of 140 was used as the baseline value before final model calibration. The calibration process was subsequently conducted, consistent with AWWA model calibration protocols [21].

Following the initial assignment of C-factors, additional hydrant fire flow tests were conducted, and the resulting data were used to refine the initial roughness values. These values were then validated using static and dynamic pressure readings from the suction and discharge sides at several pump stations. This data was then validated against historically logged telemetry data retrieved from a database maintained at the water treatment plant. Pressure data from 10 of the 14 pump stations were ultimately used in the validation process.

Model sensitivity analysis revealed that predicted pressures were more sensitive to errors in pump curves, pressure-reducing valve (PRV) settings, tank levels, and the temporal and spatial distribution of network demands than to variations in the C-factor. Consequently, the calibration effort was redirected away from C-factor values to address these more influential parameters.

Since the utility lacked reliable pump curves for modeling purposes, approximate three-point pump curves were developed using the rated horsepower from pump name-plates and measured discharge and pressure values from telemetry data. For pump stations lacking sufficient data, curves were estimated based on pumps with similar specifications and rated horsepower. In several cases, pump performance was verified by comparing hydraulic output against the filling rate of the nearest downstream storage tank.

The Martin County distribution system contains 12 PRVs that divide the network into multiple pressure zones, significantly impacting system pressures and flows. Although prescribed settings were provided by the utility, we independently inspected several PRV pits to confirm operational settings and tested downstream hydrants to verify proper PRV function. During this investigation, we discovered that measured fire flows from hydrants downstream of PRVs could change substantially during testing. In some cases, flows and pressures required over 20 min to stabilize as PRVs continued adjusting to hydrant discharge.

The distribution system was recently partitioned into 23 separate demand zones delineated by master meters. This configuration enabled estimation of each zone’s percentage of total average monthly demand through mass balance analysis. These results were used to spatially distribute the total average daily demand among the 23 demand zones. Once the percentage of total average daily demand was determined for each zone, demands were further disaggregated and assigned to individual junction nodes by counting residential and commercial connections on each pipe segment. These values were then normalized across all nodes within each zone to match the total daily demand assigned to that zone. Since master meter readings included water loss, a proportional fraction of demand at each node was assumed to represent water loss.

Diurnal demand variability was estimated through hourly mass balance analysis using flows from the water treatment plant and storage tank inflow/outflow data. The resulting demand curve used in the model is shown in Figure A1. Initially, this general curve was applied uniformly to all nodes within each demand zone, with subsequent adjustments made in some demand zones to improve agreement between observed and predicted tank water levels.

Once the Hazen-Williams roughness coefficients were finalized and initial nodal demands were assigned, a 24 h extended-period simulation (EPS) of the water distribution network was performed in KYPIPE. The resulting predicted tank levels were compared against observed levels from available telemetry data across all monitored storage tanks. In a few cases, head loss values in adjacent pipes were adjusted to achieve proper flow distribution in zones served by multiple tanks. Hydraulic model calibration was performed using tank level data recorded between 1 June at 12:00 AM and 2 June at 12:00 AM, 2024. Out of the 14 tanks in the system, five were reported by operators as non-operational, leaving nine active tanks. Of these nine (see Figure 4), reliable calibration data were available for only five tanks. Table A2 presents the calibration statistics for these five tanks, evaluated against the target criteria outlined in Table A1. Figures A2 and A3 illustrate representative calibration results, comparing observed and simulated tank levels for Tank-13 (lowest MAE and RMSE) and Tank-11 (highest MAE and RMSE). The calibration results demonstrate acceptable model performance for three of the five tanks (T-8, T-11, and T-13), which met the established target criteria. Two tanks (T-1 Buck Creek and T-2 Little Rockcastle) slightly exceeded the target thresholds due to their hydraulic interdependence, as T-2 draws from T-1 within the same demand zone. However, these tanks still exhibited relatively low MAE and RMSE values (0.377–0.481 m and 0.487–0.640 m, respectively), indicating reasonable model accuracy. Despite these localized calibration limitations, the overall model performance was deemed adequate for the water quality analysis.

**Table A2.** Tank Calibration Summary Statistics.

Tank Name	Tank ID	MAE (m)	RMSE (m)	Standard Deviation (m)
BUCK CREEK	T-1	0.377	0.487	0.722
LITTLE ROCKCASTLE	T-2	0.481	0.640	0.765
TURKEY	T-8	0.468	0.561	1.561
MARCUS WELLS	T-11	0.701	0.859	2.328
MIDDLE FORK	T-13	0.289	0.329	0.707

## Appendix B. Development of the Water Quality Model

### Appendix B.1. Modeling of Chlorine Residuals

Chlorine residuals for the Martin County water distribution system were modeled using EPANET [31]. EPANET simulates chlorine decay using a first-order decay model as shown in Equation (A1):

(A1)
$$\frac{dC}{dt}=-({\text{K}}_{\text{r}})\times \text{C}$$

where C is the concentration of chlorine (mg/L), and Kr is the composite decay coefficient (typically expressed in terms of days^−1^ or hour^−1^). The composite decay coefficient can be expanded to include the effects of bulk water decay, K_b_, the reaction of chlorine with the pipe wall, K_w_, and the mass transfer between the bulk water and the wall, represented as k_f_, as follows:

(A2)
$$\frac{dC}{dt}=-({\text{K}}_{\text{b}}+\frac{{\text{K}}_{\text{w}}\times {\text{k}}_{\text{f}}}{{\text{R}}_{\text{h}}\times ({\text{K}}_{\text{w}}\times {\text{k}}_{\text{f}})})\times \text{C}$$

where R_h_ is the hydraulic radius of the pipe. For a full pipe, the hydraulic radius is just the diameter of the pipe d, divided by 4 (i.e., ${\text{R}}_{\text{h}}=\frac{d}{4}$).

Although Equation (A2) is only explicitly expressed in terms of one water quality variable (i.e., C), the effects of other variables such as temperature, alkalinity, pH, and organic matter content are implicitly incorporated into the expanded model parameters (i.e., K_b_, K_w_, and k_f_). Therefore, these parameters can be expected to vary as a function of these variables, and thus they can be expected to vary as a function of temperature and season [32,33].

The mass transfer coefficient k_f_ can be further expressed in terms of the diffusivity D of the chemical being modeled (e.g., 1.44 × 10^−5^ cm^2^/s for hypochlorous acid, the dominant species of free chlorine), the diameter of the pipe d, and a dimensionless parameter called the Sherwood Number Sh as follows:

(A3)
$${\text{k}}_{\text{f}}=\text{Sh}\times \frac{\text{D}}{\text{d}}$$

The Sherwood Number is further dependent upon the flow conditions in each pipe. For turbulent flow conditions (Reynolds number > 2300), which is common in main distribution lines:

(A4)
$$\text{Sh}=0.023\times \text{R}{\text{e}}^{0.83}\times \text{S}{\text{c}}^{0.33}$$

Similarly, for laminar flow conditions, which may occur in dead-end pipes or during low-demand periods:

(A5)
$$\text{Sh}=3.65+\frac{\left(0.0668\times \frac{\text{d}}{\text{L}}\times \text{Re}\times \text{Sc}\right)}{1+0.04\times (\frac{\text{d}}{\text{L}}\times \text{Re}\times \text{Sc}{)}^{0.67}}$$

where Re is the Reynolds number (i.e., $\frac{\text{V}\text{d}}{n}$), Sc is the Schmidt number (i.e., $\frac{\text{d}}{\text{n}}$), L is the pipe length, and V is the pipe velocity.

In practice, the bulk decay coefficient is approximated using field samples from the water treatment plant, while the wall reaction coefficient is determined through model calibration using chlorine residual values from distribution system field sampling [34]. Both Rh and k_f_ are calculated as functions of the distribution system’s physical parameters and predicted flows in each pipe. Consequently, the composite rate coefficient K_r_ varies temporally for each pipe in the distribution system. As with all multi-parameter models, especially those that require parameter calibration, the potential exists for compensating parameter errors. However, in this case, Kb is measured directly, which only leaves Kw subject to final model calibration using actual field samples. Based on extensive past modeling experience, we do not believe that any inherent parameter errors are sufficient to jeopardize the stated objective of the current research, specifically, to demonstrate that a reduction in water loss can lead to an increase in water quality problems.

### Appendix B.2. Estimation of Chlorine Decay Coefficients in the Martin County System

To capture the effects of temperature and seasonality on both the bulk rate decay coefficient and the wall rate decay coefficient for the Martin County system, water samples were collected at the water treatment plant (for use in calculating the bulk decay coefficient) and from fire hydrants in the distribution system (for use in calibrating the wall rate decay coefficient) for both warmer (June, July, October) and colder periods (November, December, and May).

For field chlorine measurements, we used the Hach DR300 Pocket Colorimeter, which employs the N, N-diethyl-p-phenylenediamine (DPD) colorimetric method accepted by the USEPA for reporting drinking water analyses [35]. The DR300 has a measurement range of 0.02 to 2.00 mg/L in low-range mode, making it suitable for distribution systems where free chlorine concentrations typically fall within this range. The method requires adding a pre-packaged reagent to each sample bottle before measurement, with results provided within 60 s for efficient field testing. According to manufacturer specifications, the DR300 provides precision of ±0.05 mg/L at 1.00 mg/L Cl_2_ (95% confidence interval), representing approximately 5% relative error [36]. The DR300 results were also compared to chlorine measurements obtained using a Hach SL1000 Portable Parallel Analyzer colorimeter. This meter utilizes Chemkey reagent cartridge technology to reduce potential human errors in analysis. The SL1000 has a measurement range of 0.04 to 4.0 mg/L Cl_2_ [37]. Its precision is ±0.09 mg/L at 2.06 mg/L Cl_2_ (95% confidence interval) [38].

#### Appendix B.2.1. Bulk Decay

The bulk decay rate was determined for each sampling period following the EPA Free Chlorine Distribution System Influent Hold Study protocol [39]. Treated water samples were collected at the Martin County water treatment plant in chlorine-demand-free amber glass bottles with PTFE-lined caps. The bottles were pre-treated by filling them headspace-free with a 10–20 mg/L sodium hypochlorite solution, soaking for at least 24 h, and thoroughly rinsing three times with ultra-pure 18 MΩ deionized water. Sample bottles were filled headspace-free (no air bubbles) with distribution system influent water and capped tightly to prevent air exposure.

The bottles were transported to the UKY in ice chests maintained at approximately 4 °C and subsequently stored in a dark refrigerator at a stable temperature of about 7 °C. To simulate extended residence time and assess bulk chlorine reactivity, one bottle was removed each week for over four weeks for chlorine analysis. When measurements were within the stated precision of the chlorine meter, the results were averaged. If measurements exceeded the precision limits, a third reading was taken, and the outlier was excluded. Chlorine concentrations were then plotted as a function of time to develop decay curves for each sampling event.

To account for seasonal variability in chlorine decay behavior, Two distinct experimental datasets were obtained: winter–spring samples collected during the colder months (November, December, May) with a representative model of y = 1.71e^−0.0008x^, R^2^ = 0.9114, and summer–fall samples collected during warmer months (June, July, and October) with a representative model of y = 1.89e^−0.002x^, R^2^ = 0.874, both analyzed under identical laboratory conditions at 7 °C. Because the laboratory storage temperature was lower than typical distribution system conditions, the decay coefficients derived from these experimental data were adjusted using the Arrhenius equation with an activation energy of 40,000 J/mol, consistent with established literature values for chlorine decay in drinking water systems [40–42]. The Arrhenius relationship is a well-established chemical kinetics model that quantifies how reaction rates, including chlorine decay, increase with temperature due to greater molecular energy and collision frequency [40,42].

The experimental decay curves obtained under laboratory conditions (7 °C) are illustrated in Figures A4 and A5, showing the winter–spring model (K_b_ = 0.0008 h^−1^) and summer–fall model (K_b_ = 0.002 h^−1^). Temperature corrections using the Arrhenius equation were subsequently applied to these experimental models to predict chlorine behavior under typical distribution system temperatures of 16 °C for winter–spring conditions and 21 °C for summer–fall conditions. This approach yielded temperature-corrected decay coefficients of K_b_ = 0.001263 h^−1^ for winter–spring and K_b_ = 0.00488 h^−1^ for summer–fall periods. The significantly higher decay rate observed in the summer–fall experimental data, and further amplified through temperature correction, aligns with fundamental chemical kinetics principles and the findings of García-Ávila et al. [43], who observed that increasing temperature leads to higher decay coefficients and more rapid chlorine residual loss.

#### Appendix B.2.2. Wall Decay

The wall decay rate coefficient (Kw) for each sampling period was estimated by iteratively adjusting the Kw parameter in the EPANET model to achieve agreement between simulated and observed chlorine residuals at field locations. Chlorine measurements were obtained from four fire hydrants located at the extremities of the distribution system (i.e., sites M1, M2, M5, and M10). These locations served as calibration sites and are shown as squares in Figure 5. The remaining sites, shown as a triangle (the water treatment plant), diamonds (daily regulatory Cl sample locations), and circles (quarterly regulatory DBP sites), were used for subsequent model validation. Before sampling, printed chain-of-custody forms were prepared, including relevant site information and designated fields for recording field measurements.

Sample locations were selected in consultation with utility personnel to ensure both spatial coverage and operational feasibility. In some instances, utility staff accompanied the sampling team to assist with site access and coordination. At each sampling location, standard field safety protocols were followed, including the use of high-visibility vests, traffic cones, safety goggles, and protective gloves to ensure personnel safety and compliance with standard operating procedures.

### Appendix B.3. DBP (TTHM) Modeling Approach

DBPs were modeled using KYPIPE to predict the formation and distribution of TTHMs throughout the Martin County distribution system. KYPIPE typically employs a regression-based framework that allows TTHM concentrations to be expressed as a function of either chlorine demand or water age, based on available field data and system-specific characteristics [44]. For this study, we selected chlorine demand as the independent variable, as it offered a stronger empirical basis given the availability of reliable chlorine residual measurements throughout the distribution network. In addition, chlorine demand has a more direct mechanistic relationship to DBP formation processes, since TTHMs are formed because of chlorine reacting with organic matter in the bulk water [45]. Chlorine demand was defined as the difference between the initial chlorine concentration at the water treatment plant and the residual chlorine concentration observed at individual nodes within the system. Since Martin County only has one water treatment plant (designated by a triangle in Figure 5, located in the middle of the system), this facilitated the calculation of the chlorine demand at each junction node.

A functional relationship was implemented in KYPIPE using a generalized equation form that allows for various mathematical relationships between chlorine demand and TTHM formation (e.g., linear, quadratic, exponential, and polynomial relationships). Based on our calibration data, linear relationships between chlorine demand and TTHM formation were deemed adequate for the Martin County system (as long as DBP growth is not overly extrapolated beyond the range of the collected data). This approach is conceptually consistent with the work of Clark [45], who demonstrated that TTHM formation can be characterized as a function of chlorine demand, and that such relationships can be implemented using empirical regression models in distribution system simulations.

TTHM concentrations were measured at the treatment plant and the same monitoring sites (M1, M2, M5, and M10) where chlorine residuals were monitored, providing paired datasets of chlorine demand and corresponding TTHM levels for model development. To account for seasonal variability in water quality and chlorine reactivity, two separate TTHM models were developed: one for winter–spring conditions and another for summer–fall conditions. The two seasonal TTHM models are illustrated in Figures A6 and A7, respectively. For the winter–spring model, the linear relationship between chlorine demand and TTHM formation was established using 8 data points collected between May 2023 and May 2025. The resulting regression relationship yielded an R^2^ value of 0.6729, which meets the target threshold (see Table A1). Similarly, the summer–fall model was developed using 8 measurements collected between July 2022 and June 2024, achieving an R^2^ value of 0.8419, indicating good model performance according to the evaluation criteria. Both seasonal models demonstrated adequate predictive capability for simulating TTHM formation throughout the distribution system.

## Appendix C. Field Sampling, Calibration, and Validation Procedures

### Appendix C.1. Chlorine Sampling Procedures

For hydrant field sampling, specialized equipment such as hydrant wrenches were used to safely operate the hydrants. Extra care was taken when opening and closing each hydrant to prevent transients in the distribution system. Each hydrant was flushed for at least five minutes to remove debris or sediment from the hydrant barrel and ensure representative samples from the distribution system. During flushing, precautions were taken to prevent water from affecting traffic or causing flooding of surrounding properties. Following flushing, the hydrant was temporarily closed, and a short segment of valved 25 mm PVC pipe was attached to one of the 65 mm nozzles using a customized hydrant cap. The hydrant was then reopened and pressurized, with the PVC valve used to regulate water flow and facilitate sample collection. After sampling completion, the hydrant was closed, the sampling cap removed, and the regular hydrant cap reinstalled.

Before analysis, sample cells were thoroughly rinsed three times with the sample and filled to the 10 mL mark for the DR300 or the designated fill line for the SL1000. Sample vials were cleaned with Kimwipes to remove fingerprints or liquids that could interfere with readings. For the DR300, the colorimeter was manually zeroed before each measurement using a blank sample. A chlorine reagent packet was then added to the sample, mixed thoroughly for 20 s, and analyzed within one minute. The SL1000 performed automatic calibration using Chemkey reagent cartridges. All sampling was conducted using proper safety precautions, including powder-free gloves and safety glasses, with special care when handling DPD reagents due to potential eye irritation.

A comprehensive QA/QC protocol ensured measurement reliability and accuracy. While manufacturers pre-calibrated both instruments, the SL1000 was used to verify DR300 chlorine results. At each location, a minimum of two independent measurements were taken for both free and total chlorine using the DR300, with verification that free chlorine readings were always less than total chlorine readings. When the absolute difference between duplicate free chlorine readings exceeded ±0.05 mg/L or when the relative difference exceeded ±10% (whichever was greater), a third measurement was performed. Erroneous readings were identified as those that: (1) exceeded the instrument’s measurement range (>2.00 mg/L for DR300 in low-range model), (2) showed free chlorine values greater than total chlorine values, (3) differed from the other two readings by more than ±0.10 mg/L when three measurements were taken, or (4) showed instrument error messages or unstable readings. When three measurements were taken, any extreme values violating these criteria were noted and excluded, and the average of the remaining two values was recorded. Results were then compared against SL1000 readings, and measurements were repeated when the absolute difference between instruments exceeded ±0.10 mg/L or ±15% (whichever was greater). The QA/QC protocol described here for field sampling is consistent with the measurement replication approach used for bulk decay experiments, as discussed in Appendix B.2 where duplicate measurements were also collected for each measurement. The more extensive QA/QC documentation for field sampling reflects the additional complexity of field conditions, including the need for cross-instrument verification and management of multiple sources of field variability.

### Appendix C.2. DBP (TTHM) Sampling Procedures

DBP samples were collected following USEPA Method 551.1 protocols [46]. Before sampling, the area was inspected to ensure no chlorine or bleach-containing cleaning products were near the sampling location. All sampling materials (containers, blanks, etc.) were prepared in advance, and water was flushed from the sampling hydrant until a stable temperature was reached. The flow from the sampling pipe was then reduced to a trickle (approximately pencil thickness), and personnel donned new powder-free nitrile gloves, being careful not to touch the faucet handles, outside of plastic bags, or other potentially contaminating surfaces.

Two separate 40 mL volatile organic compounds (VOC) vials, pre-preserved with phosphate buffer and ammonium chloride dechlorinating agent, were filled carefully to prevent overflowing, which would dilute the preservatives. The vials were filled just to the top, and then the cap was filled with water. A few drops were then added to the cap until the water mounded at the top of the vial due to surface tension. The vials were capped carefully, inverted, and checked for air bubbles, which were eliminated by adding additional water if present. Vials were sealed, labeled, and placed in labeled plastic bags before being transported on ice packs in a cooler to maintain stability.

Comprehensive QA/QC protocols were followed throughout the sampling process. For every five sample collections, a field blank was prepared by briefly opening a 40 mL VOC vial containing ultrapure water, then recapping it after 30–60 s and checking for air bubbles. Duplicate samples were also collected as a precaution, ensuring the analysis could still be conducted if the initial samples were compromised. Sample temperature was continuously monitored during transport, with coolers and ice packs maintaining temperatures at or below 4 °C. Samples were transported to the UKY plant and soil sciences laboratory and analyzed within 14 days to comply with regulatory holding times. A detailed chain of custody was maintained for all DBP samples requiring laboratory analysis to ensure data integrity from collection through final processing.

Samples were extracted and analyzed by liquid–liquid extraction followed by gas chromatography-electron capture detection (GC-ECD), following the procedures outlined in EPA method 551.1 (Agilent 8890, Santa Clara, CA, USA). The method involves simultaneous quantification using a primary column and a confirmation column, of differing chromatographic selectivity. Calibration was performed using NIST-traceable standards and surrogates, and confirmation of the calibration curve using a standard from an independent lot number (Absolute Standards, Hamden, CT, USA). Each sample was spiked with a surrogate (decafluorobiphenyl), and blanks, duplicates, and spike recovery samples were included with each analytical batch.

### Appendix C.3. Model Calibration Procedures

#### Appendix C.3.1. Chlorine Residual Calibration

Once free chlorine measurements were collected from selected field sites during both summer and winter sampling events, EPANET was used to simulate chlorine concentrations at each location. For each simulation, the hydraulic boundary conditions for the specific sampling day were set (i.e., total demand, tank levels, pump status, etc.), and the observed chlorine concentration at the treatment plant was entered as the initial boundary condition.

The observed Kb value was input for each model, and an initial Kw value was assumed to begin the calibration process. The model was run for a 1000 h (~6 weeks) simulation period under the assumption of repeating daily demand patterns and pump schedules. This extended run time was necessary to allow the chlorine concentrations in the system to approach a quasi-steady state. For calibration purposes, the average of this oscillation range was used as the predicted steady-state value.

The modeled average chlorine concentrations at each monitored hydrant location (see Figure 5) were then compared to the observed field measurements, as presented in Table A3. Table A3a shows winter–spring model calibration results, while Table A3b presents summer–fall model performance. Where observed chlorine values fell outside the predicted range, Kw values for pipes leading to each sampling site (local Kw values) were iteratively adjusted to improve model agreement. The summer–fall model demonstrated better performance, with an MAE of 0.028 mg Cl/L and individual site errors ranging from 0.01 to 0.06 mg Cl/L. The winter–spring model showed slightly higher prediction errors with an MAE of 0.055 mg Cl/L and errors ranging from 0.01 to 0.11 mg Cl/L. Both models achieved excellent overall accuracy, with MAPEs of 2.8% and 5.40% for summer–fall and winter–spring conditions, respectively. Both seasonal models meet the established target criteria outlined in Table A1, with MAPE values well below 25% and demonstrating low error and appropriate model calibration performance. The global Kw values used for both models are also shown in both tables.

**Table A3.** Winter–Spring model calibration results for chlorine residuals in the Martin County distribution system (**a**); Summer–Fall model calibration results for chlorine residuals in the Martin County distribution system (**b**).

(a)					
Site ID	Date	Measured Cl (mg/L)	Predicted Cl (mg/L)	Absolute Error (mg/L)	Percent Error (%)
M1	30 May 2023	1.2	1.19	0.01	0.83
M2	30 May 2023	1.11	1.00	0.11	9.91
M5	30 May 2023	1.25	1.20	0.05	4.00
M10	30 May 2023	0.73	0.68	0.05	6.85
Kw = −0.04 h^−1^				MAE = 0.055 mg/L	MAPE = 5.40%
(b)					
Site ID	Date	Measured Cl (mg/L)	Predicted Cl (mg/L)	Absolute Error (mg/L)	Percent Error (%)
M1	19 June 2024	1.43	1.41	0.02	1.40
M2	19 June 2024	1.21	1.15	0.06	4.96
M5	19 June 2024	0.61	0.60	0.01	1.64
M10	19 June 2024	0.65	0.63	0.02	3.08
Kw = −0.02 h^−1^				MAE = 0.028 mg/L	MAPE = 2.80%

#### Appendix C.3.2. TTHM Calibration

Following the completion of chlorine residual model calibration, TTHM formation relationships were applied to predict DBP concentrations throughout the distribution system. The regression equations (see Figures 6 and 7), previously established using observed chlorine and observed TTHM field measurements, relate chlorine demand to TTHM formation for winter–spring and summer–fall conditions. To apply these TTHM regression relationships, chlorine demand was first calculated at each monitoring site using the calibrated chlorine residual concentrations. The initial chlorine concentration at the treatment plant was 1.9 mg/L for winter–spring conditions and 1.7 mg/L for summer–fall conditions during the respective sampling campaigns. These values were obtained from the continuous HACH Chlorine Analyzer located in the Martin County water treatment laboratory and were field checked using both the DR300 and SL1000 meters. The values obtained from all three meters were within the stated precision of the meters (i.e., 0.05 mg/L). Each field sample was analyzed using the same instruments and protocols described in Appendix C.1.

The predicted TTHM concentrations were subsequently compared to the observed TTHM field measurements collected at the monitoring sites. Table A4 presents the calibrated results for both seasonal models, showing measured and predicted TTHM concentrations at each monitoring site along with absolute errors and model performance metrics. Seasonal differences in model performance were evident, with the winter–spring model demonstrating better performance (MAE = 0.002 mg TTHM/L, MAPE = 3.3%, error range: 0.000–0.003 mg TTHM/L) compared to the summer–fall model (MAE = 0.017 mg TTHM/L, MAPE = 23.9%, error range: 0.004–0.023 mg TTHM/L). The winter–spring model performed better at all sites, showing individual percent errors below 6%, while the summer–fall model exhibited greater variability, with three sites (M1, M5, and M10) showing higher prediction errors. The increased prediction variability during summer–fall reflects the temperature-dependent nature of chlorine decay and DBP formation reactions, as well as potential seasonal changes in source water quality and organic precursor concentrations. Despite these seasonal differences, both models meet the established performance criteria outlined in Table A1, with the summer–fall model’s MAPE remaining within acceptable limits, supporting the use of dual-season calibration strategies for distribution system DBP modeling.

**Table A4.** Winter–Spring model performance for total trihalomethane (TTHM) prediction in the Martin County distribution system (**a**); Summer–Fall model performance for total trihalomethane (TTHM) prediction in the Martin County distribution system (**b**).

(a)						
Date	Site	Predicted Chlorine Demand (mg/L)	Measured TTHM (mg/L)	Predicted TTHM (mg/L)	Absolute Error (mg/L)	Percent Error (%)
30 May 2023	M1	0.71	0.056	0.055	0.001	1.79
	M2	0.90	0.055	0.058	0.003	5.45
	M5	0.70	0.052	0.055	0.003	5.77
	M10	1.22	0.062	0.062	0.000	0.00
					MAE = 0.002 mg/L	MAPE = 3.3%
(b)						
Date	Site	Predicted Chlorine Demand (mg/L)	Measured TTHM (mg/L)	Predicted TTHM (mg/L)	Absolute Error (mg/L)	Percent Error (%)
19 June 2024	M1	0.29	0.062	0.045	0.017	27.42
	M2	0.55	0.066	0.062	0.004	6.06
	M5	1.10	0.073	0.096	0.023	31.51
	M10	1.07	0.072	0.094	0.022	30.56
					MAE = 0.017 mg/L	MAPE = 23.9%

### Appendix C.4. Model Validation Procedures

#### Appendix C.4.1. Chlorine Residual Validation

The seasonal chlorine residual models were validated using field data reported in KY’s Monthly Operating Reports (MORs) from the treatment plant. Although MORs contain observed chlorine concentrations at various points in the distribution system, the results are aggregated and labeled by directional zones (North, South, East, and West), with monitoring locations rotated monthly. Consequently, while some sampling sites are known, the exact junction IDs are not consistently reported across months. To align model outputs with the MOR reporting structure, representative model junctions (V1, V2, V3, and V4) were selected to reflect conditions in each directional zone. These validation points were chosen based on spatial proximity to known sites, hydraulic similarity, and network topology, ensuring that model outputs reasonably approximated the reported field conditions.

For each validation run, the calibrated Kw values were held constant, while average monthly demands were used to simulate operating conditions. The predicted chlorine residuals were then compared to the observed MOR values. Table A5 summarize the validation results, including observed and predicted concentrations, as well as associated absolute and percent errors for both seasons. The winter–spring model was validated using data from November 2023, and the summer–fall model using data from August 2024. Both models demonstrated good performance, with the winter–spring model achieving an MAE of 0.088 mg/L and MAPE of 7.9%, compared to the summer–fall model with an MAE of 0.080 mg/L and MAPE of 8.9%. Interestingly, while the winter–spring model showed slightly better percentage accuracy, the summer–fall model exhibited a lower mean absolute error. Both validation results fall well within acceptable performance criteria (see Table A1), demonstrating reasonable seasonal accuracy and supporting the transferability of the calibrated models to independent datasets. Figure A8 provides a visual comparison of observed and predicted values at each validation site, reinforcing the quantitative accuracy of the models.

**Table A5.** Winter–spring model validation results for chlorine residuals in the Martin County distribution system (**a**); summer–fall model validation results for chlorine residuals in the Martin County distribution system (**b**).

(a)					
Date	Site	Observed (mg/L)	Predicted (mg/L)	Absolute Error (mg/L)	Percent Error (%)
November 2023	V1	0.97	0.96	0.01	1.03
	V2	1.28	1.40	0.12	9.38
	V3	1.24	1.16	0.08	6.45
	V4	0.96	1.10	0.14	14.58
				MAE = 0.088 mg/L	MAPE = 7.9%
(b)					
Date	Site	Observed (mg/L)	Predicted (mg/L)	Absolute Error (mg/L)	Percent Error (%)
August 2024	V1	0.86	0.95	0.09	10.47
	V2	1.21	1.23	0.02	1.65
	V3	1.05	1.20	0.15	14.29
	V4	0.64	0.70	0.06	9.38
				MAE = 0.080 mg/L	MAPE = 8.9%

#### Appendix C.4.2. TTHM Model Validation

The seasonal TTHM formation models were validated using field measurements from KY regulatory monitoring locations, specifically the Tug Fork and Meat House sites (see Figure 5), which represent critical compliance points in the distribution system. Validation was performed using data from 27 November 2023, for the winter–spring model and 26 August 2024, for the summer–fall model, corresponding to the dates reported by the state for their quarterly DBP measurements.

It should be noted that the water treatment plant is required by KY regulations to monitor DBPs quarterly at designated compliance locations. While individual TTHM readings exceeding 0.080 mg/L may raise concern, regulatory violations are determined based on quarterly locational running annual averages (LRAA) calculated from four consecutive quarters of data, rather than single sample measurements.

For TTHM validation, the previously calibrated chlorine residual models were run using actual demands for the validation periods. The predicted chlorine residuals were then used to calculate chlorine demand at each monitoring location, which served as input to the seasonal TTHM models. The resulting predicted TTHM concentrations were compared against field measurements reported to the state for the respective validation periods.

The validation approach maintained model integrity by using the same calibrated parameters and regression relationships developed during the initial calibration phase, ensuring that the validation represented true model performance rather than parameter adjustment to fit validation data. This methodology provides confidence in the models’ ability to predict TTHM formation under actual operating conditions and supports their use for proactive compliance management and system optimization.

The TTHM model validation results, shown in Table A6 and Figure A9, highlight noticeable seasonal differences in performance. The summer–fall model showed good predictive accuracy (MAPE = 11.5%), with reasonable agreement at both sites. In contrast, the winter–spring model had lower accuracy (MAPE = 22.6%), mainly due to underprediction at the Meat House location. This difference aligns with the stronger calibration fit in the summer–fall model (R^2^ = 0.84 vs. 0.67), indicating a more consistent chlorine demand–TTHM relationship during warmer months. Both validation measurements at Tug Fork and Meat House exceeded or approached the MCL of 0.080 mg/L for TTHMs, with observed concentrations ranging from 0.074 to 0.123 mg/L. While individual sample exceedances do not constitute regulatory violations, these results highlight the importance of accurate DBP modeling for proactive compliance management. Overall, both seasonal models together achieved reasonable validation accuracy (combined MAPE = 17.0%, MAE = 0.016 mg/L), supporting their use for TTHM prediction in the distribution system.

**Table A6.** Validation results for seasonal total trihalomethane (TTHM) formation models at regulatory monitoring sites in the Martin County distribution system.

Date/Model	Site	Observed (mg/L)	Predicted (mg/L)	Absolute Error (mg/L)	Percent Error (%)
27 November 2023 (Winter–Spring model)	Tug Fork	0.074	0.062	0.012	16.22
	Meat House	0.083	0.059	0.024	28.92
				MAE = 0.018 mg/L	MAPE = 22.6%
26 August 2024 (Summer–Fall model)	Tug Fork	0.121	0.105	0.016	13.22
	Meat House	0.123	0.111	0.012	9.76
				MAE = 0.014 mg/L	MAPE = 11.5%
